# Integrative whole-genome and transcriptome analysis of HER2-amplified metastatic breast cancer

**DOI:** 10.1186/s13058-023-01743-z

**Published:** 2023-11-15

**Authors:** Noortje Verschoor, Marcel Smid, Agnes Jager, Stefan Sleijfer, Saskia M. Wilting, John W. M. Martens

**Affiliations:** Department of Medical Oncology, Erasmus MC Cancer Institute, University Medical Center Rotterdam, Dr. Molewaterplein 40, 3015 GD Rotterdam, The Netherlands

**Keywords:** Breast cancer, Metastatic tissue, HER2 positive, WGS, RNA sequencing

## Abstract

**Background:**

In breast cancer, the advent of anti-HER2 therapies has made HER2+ tumors a highly relevant subgroup. However, the exact characteristics which prohibit clinical response to anti-HER2 therapies and drive disease progression are not yet fully known. Integrative whole-genome and transcriptomic sequencing data from both primary and metastatic HER2-positive breast cancer will enhance our understanding of underlying biological processes.

**Methods:**

Here, we used WGS and RNA sequencing data of 700 metastatic breast tumors, of which 68 being HER2+, to search for specific genomic features of HER2+ disease and therapy resistance. Furthermore, we integrated results with transcriptomic data to associate tumors exhibiting a HER2+-specific gene expression profile with *ERBB2* mutation status, prior therapy and relevant gene expression signatures.

**Results:**

Overall genomic profiles of primary and metastatic HER2+ breast cancers were similar, and no specific acquired genomics traits connected to prior anti-HER2 treatment were observed. However, specific genomic features were predictive of progression-free survival on post-biopsy anti-HER2 treatment. Furthermore, a HER2-driven expression profile grouped HER2-amplified tumors with ERBB2-mutated cases and cases without HER2 alterations. The latter were reported as ER positive in primary disease, but the metastatic biopsy showed low ESR1 expression and upregulation of the MAPK pathway, suggesting transformation to ER independence.

**Conclusions:**

In summary, although the quantity of variants increased throughout HER2-positive breast cancer progression, the genomic composition remained largely consistent, thus yielding no new major processes beside those already operational in primary disease. Our results suggest that integrated genomic and transcriptomic analyses may be key in establishing therapeutic options.

**Supplementary Information:**

The online version contains supplementary material available at 10.1186/s13058-023-01743-z.

## Background

Amplification of the *ERBB2* gene, encoding human epidermal growth factor receptor 2 (HER2), occurs in approximately 15% of primary breast cancers (PBC) and causes overexpression of the protein kinase receptor HER2. Among patients with a HER2-amplified (HER2-positive/HER2+) tumor, targeted monoclonal antibodies binding and blocking this receptor of which trastuzumab is the most commonly administered proved to be very effective [1], resulting in a major increase in survival in this inherently aggressive breast cancer subtype [2]. As a result, HER2+ tumors constitute a distinct subgroup from a clinical perspective. In contrast, whole-genome sequencing (WGS) and whole-exome sequencing (WES) data from PBC revealed that HER2+ tumors are heterogeneous with regard to their molecular characteristics and often cluster with all other subtypes [3].

At the protein level, overexpression of HER2 in metastatic breast cancer (MBC) compared PBC to is rather stable [4]. Despite very pronounced and durable responses to regimens containing HER2-targeted agents, resistance is not uncommon. For example, around 40% of HER2 + MBC patients treated with dual HER2 blockade combined with docetaxel in the first line of therapy according to current clinical guidelines experience progression within the first year of treatment [5, 6]. The stable overexpression of HER2 despite disease progression suggests that mechanisms other than those involving downregulation or inactivation (e.g. via mutation) of the receptor are responsible for resistance. Several alternative trastuzumab-related mechanisms have been proposed in the literature, including activating *ERBB2* mutations, alternate receptor cleavage and epitope masking, but also alterations in the downstream pathways and activation of alternative signaling pathways [7, 8]. Furthermore, therapy resistance might be caused by resistance against the cytotoxic backbone, given the observation from clinical data that admission of trastuzumab beyond progression has clinical benefit [9, 10].

Results from targeted sequencing of a panel of genes in both primary and metastatic HER2+ tumors revealed that there was a significant enrichment of mutations in the MAPK pathway in metastatic tumors indicating that in response to treatment with targeted therapy, a proportion of HER2+ cancers switch from *PI3K/AKT* signaling to *MEK/ERK* signaling [11]. As opposed to targeted data, genome wide data might yield even more insight into resistance mechanisms, but to date there is no report on a large and clinically annotated cohort of metastatic HER2+ MBC.

The present study therefore uses WGS data from metastatic breast cancer and aims to compare the observed alterations and mutational processes with publicly available WGS data of unpaired primary breast cancer. Additionally, we investigate the associations between observed alterations in metastatic cases and response to HER2-targeted therapy to gain more insight into resistance mechanisms. Finally, we perform integrative genomic and transcriptomic analyses to evaluate downstream HER2-driven signaling in HER2-amplified and non-amplified tumors.

## Methods

### Study design and patients

WGS and RNA sequencing data were obtained from metastatic lesions of patients with metastatic breast cancer participating in the Center for Personalized Cancer Treatment (CPCT) consortium study (CPCT-02, NCT01855477), which was approved by the medical ethics committee of the University Medical Center Utrecht, the Netherlands. Details about the whole cohort have been described before [12]. Patients with metastatic breast cancer (*n* = 878) from which WGS and/or RNA sequencing data were available were included. WGS and RNA sequencing workflows have been previously described [13]. Biopsies of the primary tumor (*n* = 74) and sequential biopsies (*n* = 78) were excluded. Furthermore, when analyses called for stratification by ER status, samples with unknown primary ER status were excluded (*n* = 26) (flowchart in Additional file 1: Figure S1). Tumor responses to the treatment following biopsy were measured according to RECIST v1.1 every 8–12 weeks, and best overall response was defined as complete response (CR), partial response (PR), stable disease (SD) or progressive disease (PD) [14]. Progression-free survival (PFS) was defined as the time between start therapy and the response date, with PD scored as event and other responses (CR, PR and SD) as censored (using the latest response date known for that patient).

### Cataloging somatic changes

Processing of raw sequencing data of matched tumor and normal, and identification of somatic events (nucleotide and structural variants) was performed as described before [12]. Mutational signatures (SBS, DBS and ID, COSMIC v3 [15]) were called using R package MutationalPatterns v1.10.0 [16], and for SV signatures, we used Sigminer v2.1.3 [17]. The tumor’s copy number profile was estimated by using the B-allele frequency, read depth and SVs, as previously described [12]. Recurrent CNV regions were identified using GISTIC v2.0.23, with settings as described previously [18, 19].

### RNA sequencing

RNA was isolated and processed as previously described [19]. Raw sequencing data were mapped using STAR (v2.6.1d) [20], and Sambamba (v0.7.0) [21] was used to mark duplicates and index the resulting BAM files. Gene annotation was derived from GENCODE Release 30 (https://www.gencodegenes.org/), and raw read counts were obtained with featureCounts (v1.6.3) [22] and normalized using GeTMM [23].

### Assignment of HER2 status

The HER2 status was established using data of the metastatic lesion. First, for the samples with available RNAseq data, *ERBB2* expression was used in a mixed-model estimation and plot a bimodal histogram (Additional file 1: Figure S2a). The cross-point of the estimated curves was used as expression cut point, which was used to make an intermediate high/low call. This intermediate call was used as grouping and associated with the copy number (CN) of the same samples in a ROC analysis (Additional file 1: Figure S2b). A CN threshold of 9.954 was the optimal cut point (sensitivity of 97.8% and specificity of 98.6%) which was then used to call all samples, including those without RNAseq data available. All samples that were above this specified threshold were HER2+ in our analysis and all other samples were HER2− (as stated in Additional file 1: Figure S1). For comparison with primary HER2+ tumors, the BASIS cohort was used [24]. Here, HER2 status was assigned by a pathologist, according to clinical guidelines.

### Calculating a risk score for PFS

A model of 5 characteristics; *PIK3CA* and *CDK12* mutation, mutational signature DBS3, copy number region Amp-peak 6 (copy number gain on chr8p11.23, wide peak chr8:37467027–37502588 with ZNF703 as the closest gene), and number of prior therapies, were combined into a single risk score. *PIK3CA*, *CDK12* and Amp-peak 6 were scored as 1 when mutated/amplified. DBS3 was scored as 1 when the contribution in the respective sample was above the median contribution across all samples. The number of prior therapy lines was scored as 0.25 for patients with no prior lines, as 0.5 for 1 prior line, 0.75 for 2 or 3 lines and 1 for 4 or more prior lines of therapy. A high risk was assigned to patients with at least a score of 2.25 (optimal cut point). For the validation cohort from Smith et al. [11], DBS3 and prior lines were not available. For the Amp-peak 6, the ‘region limit’ coordinates (chr8:32280146-47560553) provided by GISTIC were used to find CN segments within these limits that had a CN > 1.5 in the validation data (using the publicly available ‘data_cna_hg19.seg’ file) and labeled samples having such a region as Amp-peak 6 positive. Events in *PIK3CA*, *CDK12* and Amp-peak 6 appeared mutually exclusive in the metastatic samples of that cohort, so high risk for the validation samples was assigned based on the presence of either one of the three characteristics.

### HER2-driven expression profile

To obtain genes that are associated with an active HER2 expression profile, samples were grouped in 4 categories using 2 characteristics: (1) HER2+ or HER2− and (2) prior anti-HER2 therapy (given prior to the biopsy) yes or no. The reason to include prior anti-HER2 therapy as group is that those samples were under selective pressure and potentially implemented alternative ways to keep an active HER2 pathway, independent of *ERBB2* amplification. A Kruskal–Wallis test was performed on the 4 groups, and genes were selected with a *p* value < 0.001. Genes on chromosome 17 were excluded as these are potential passenger events, higher expressed due to co-amplification with *ERBB2*. This yielded 878 genes (Additional file 1: Table S2); next, expression levels were median centered and used to create a correlation matrix of sample vs sample. This matrix of correlation coefficients was subsequently used for hierarchical clustering. To verify findings, publicly available microarray data of primary tumors of 867 BC patients were used; cases had comparable clinical background (lymph node negative, not adjuvant chemo/hormonal treated) and were all analyzed on the same microarray platform/chip type (Gene Expression Omnibus, accession codes GSE2034, GSE5327, GSE2990 and GSE7390). Raw.cel files were downloaded and processed with fRMA, and batch effects were corrected using ComBat.

### Statistical analysis

Categorical data were analyzed using a Pearson’s Chi-squared test or Fisher’s exact test (in case of too few expected events), and continuous variables were evaluated using either a Mann–Whitney U test (MWU) or a Kruskal–Wallis (KW) test depending on the number of categories. Cox’s proportional hazards model was used to identify items associated with PFS. The forward selection procedure to include items in a multivariable model consisted of including the most significant univariate item and only keeping the next significant item if it remained significant in the multivariable model. All statistical tests were two-sided and considered statistically significant when *p* < 0.05. Stata 13.0 (StataCorp) and R (v4.0.3) were used for the statistical analyses. Multiple testing using the Hochberg procedure to correct *p* values was applied when necessary. The statistical test used is specified throughout the results section.

## Results

### Cohort characteristics

A cohort of 736 fresh frozen metastatic lesions from unique BC patients was available for analyses (Additional file 1: Figure S1). The ER status was known for the primary disease in 700 cases and showed 568 ER + cases (81%). The biopsies of the metastases were mainly taken from the liver (45.2%), lymph nodes (19.6%) and bone (12.2%). Using the WGS data of this cohort, somatic single- and multiple-nucleotide variants (SNV/MNV), insertions/deletions (InDels), copy number variants (CNV) and structural variants (SV) were obtained. Based on CNV data of *ERBB2,* 68 MBC cases were considered as HER2-amplified (HER2+, see methods and Additional file 1: Figure S2).

The tumor mutational burden (TMB, the number of SNV/MNV/InDels per Megabase) of the HER2+ cases ranged from 0.65 to 32.51 with a median of 4.37 (95% confidence interval (CI) of 3.54–5.17). The number of SVs ranged from 78 to 2181, showing a median of 599 (95% CI 494.33–706.01). In line with the literature [25], the TMB and number of SVs were significantly higher in HER2+ versus HER2− tumors (MWU *p* = 0.0024 and *p* = 6.977e−12, respectively); however, this observation was partly driven by ER status as differences were less pronounced when only investigating ER-negative tumors (ER−/HER2+ vs ER-/HER2: MWU *p* = 0.64 and *p* = 0.011 for TMB and SV, respectively). However, when considering a cutoff of 10 mutations per MB, which defined as TMB-high in the literature [25], a small proportion of 6% of samples adhered to this definition. This was not significantly different from the proportion of TMB-high samples in the ER+- and triple-negative subtypes (TMB > 10/MB in 11% and 12%, respectively, Chi-square test *p* = 0.37).

### Metastatic HER2-positive tumor tissue is comparable to HER2-positive primary breast cancer

To evaluate potential differences between unpaired primary and metastatic HER2+ tumor tissue, several genomic characteristics were compared: the TMB and number of SVs, putative driver mutations (here defined as amino acid changing events), mutational signatures (including Single and Double Base Substitutions, Insertion/Deletion (Indel) and SV signatures, respectively, SBS, DBS, ID and SVsig). The frequencies of 68 HER2+ MBC cases were compared to the 73 HER2+ PBC cases from the BASIS cohort [24] (showing a balanced proportion of 37% ER-negative cases for both cohorts, Fisher exact test *p* = 0.95).

**Fig. 1:**
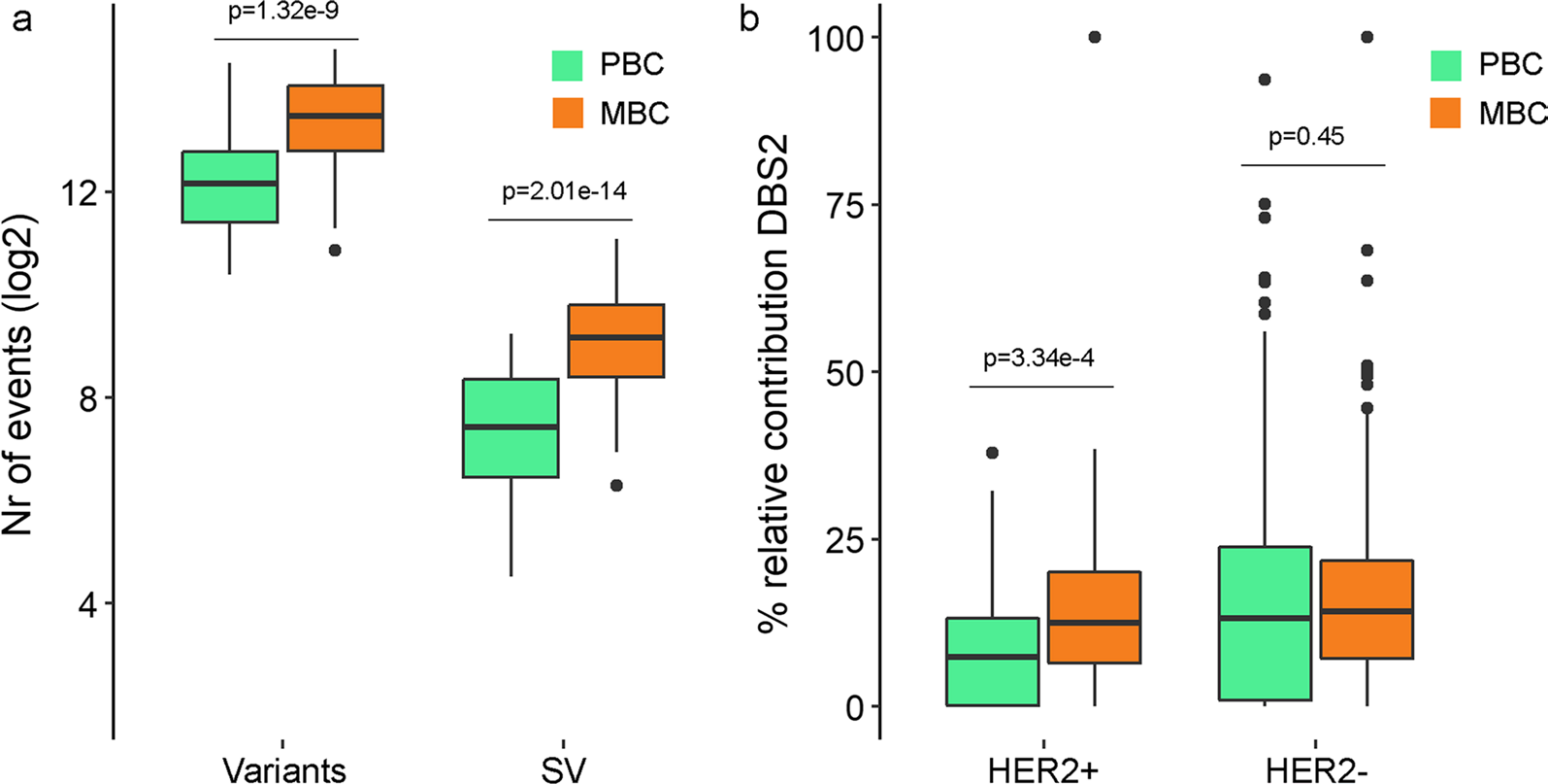
Differences between MBC and PBC. Levels and frequencies in unpaired PBC (green) and MBC (orange) for **a** total number of variants, being single-nucleotide variants and indels, and number of SVs, **b** % of relative contribution of the DBS2 mutational signature in HER2+ (left) and HER2− (right), showing that enrichment of the DBS2 signature in metastatic tumors is subtype-specific. *p* values derived from FDR-corrected MWU tests

In metastatic lesions, the median number of somatic nucleotide variants (including Indels) was 11,845.5 (interquartile range (IQR): 7364.5–19,398) and the median number of SVs was 573.5 (IQR: 339–912.5) which both were significantly higher than the number of events in HER2+ PBC (median, IQR and MWU for somatic nucleotide variants were 4702 (2935–7481) *p* = 1.318e−9 and for SVs were 172 (88–329), *p* = 2.014e−14; Fig. 1a).

Due to this significantly higher overall TMB in MBC, differences in mutation frequencies of individual genes between unpaired MBC and PBC were investigated taking TMB into account in a multivariate model. After correcting for multiple testing, besides a higher TMB (regression coefficient (95%CI) 0.027 (0.009–0.044), *p* = 0.003) a higher frequency of *TP53* mutations was observed in MBC tissues (63% vs 36%, false discovery rate (FDR) corrected Fisher exact test *p* = 0.028), regardless of ER status (regression coefficient (95%CI) 0.266 (0.109–0.423), *p* = 0.001). No other significant differences in mutation frequency were observed. Next, a post hoc analysis of *TP53* mutation frequency differences between MBC and PBC in other clinical subtypes—ER positive, HER2 negative and triple negative—also showed enriched *TP53* mutations in MBC compared to PBC (for both subtypes MWU *p* < 0.0001), indicating enrichment of *TP53* mutations was not specific for the HER2+ subtype. Of note, *ERBB2* mutations were numerically higher in HER2+ MBC (9 out of 68) compared to HER2+ PBC (2 out of 73, uncorrected Fisher exact *p* = 0.027), but this failed to reach statistical significance after correcting for multiple testing (*p* = 0.35).

Next, differences in predefined COSMIC mutational signatures [15] between HER2+ MBC and HER2+ PBC were compared for ER+- and ER-negative cases separately. Out of all possible signatures, those with at least 10% contribution in at least 10% of the MBC samples were evaluated (*n* = 26, see Additional file 1: Table S1). Only DBS2 appeared to be enriched in HER2+ MBC compared to HER2+ PBC regardless of ER status (FDR corrected MWU *p* = 0.0057, Fig. 1b). Though significant higher, the median contribution DBS2 in MBC samples of 13.5% (vs 7.4% in PBC) appeared modest. In summary, besides a general increase in the overall number of somatic changes, no evidence was found for specific somatic changes associated with progression of HER2+ disease.

Finally, potential genomic scarring due to anti-HER2 treatment administered prior the biopsy was evaluated. HER2+ samples with prior anti-HER2 therapy (*n* = 32) were compared to HER2+ samples with no prior treatment (*n* = 20). Analyzing mutation frequencies and mutational signatures, with and without stratification for ER status, showed no significant differences after multiple testing correction.

### Genomic features of therapy resistance

Next, WGS data were explored for factors that could predict response to HER2-targeted therapy. To this end, genomic characteristics of the metastasis were associated by Cox regression analysis to response in patients who received anti-HER2 therapy after tissue biopsy (*n* = 69, of which 44 ER+, 22 ER negative and 3 with unknown primary ER status. These patients were not necessarily HER2+ according to our genomic pipeline). For 33 patients (48%), this was the first line of therapy for metastatic disease. Treatment following biopsy included trastuzumab-based therapy (*n* = 66), T-DM1 (*n* = 2) and lapatinib (*n* = 1). Survival was defined from the start of treatment to radiological progression. Otherwise, patients were censored at the last radiological evaluation. Items tested in univariate Cox regression analysis (95 items in total) were the number of mutations (all somatic nucleotide changing variants), number and type of structural variants, WGS-estimated genome ploidy, mutational signatures (those with at least 10% contribution in at least 10% of samples), whole-genome duplication (WGD) and chromothripsis status, genes mutated (amino acid changing variants) and recurrent CNV regions (identified using GISTIC). Furthermore, ER and HER2 status, as well as the number of prior therapy lines were included (full list in Additional file 1: Table S1) In total, 13 items were significantly associated with progression-free survival time in univariate analyses (PFS, Cox regression *p* < 0.05), of which 5 remained significant in a multivariable model following forward selection: the number of lines of prior treatment, mutations of *PIK3CA* or *CDK12*, gain of chromosome 8p11.23 and a high contribution of gene signature DBS3 (Table 1).

**Table 1:** Univariable and multivariable Cox model for progression-free survival

	Univariable		Multivariable				
	Item	*p* value	HR	95% CI	*p* value	Status	*n* (%)
Clinical	Lines of prior treatment	0.0278	1.43	1.02–1.99	0.0360	0	18 (26)
	(adjuvant treatment as one line if applicable)					1	25 (36)
						2–3	13 (19)
						≥ 4	13 (19)
Mutation	PIK3CA	0.0086	5.53	2.41–12.71	5.60E–05	Mutated	27 (39)
	PIK3CA or ERBB2	0.0100					
	CDK12	0.0135	3.47	1.12–10.73	0.0310	Mutated	9 (13)
	HMCN1	0.0200					
	CACNA1G	0.0285					
Signatures	DBS3	0.0009	18.07	5.71–57.17	8.50E−07	> 10% contribution	8 (12)
	SBS18	0.0210					
	SBS39	0.0239					
	SBS40	0.0417					
	SV6	0.0407					
Copy numbers	Gain 8p11.23	0.0105	3.91	1.91–8.03	0.0002	Present	24 (35)
	WGD	0.0286					

*HR* hazard ratio, *CI* confidence interval

*Items that reached statistical significance (*p* < 0.05) were used in a multivariable Cox regression to model outcome on anti-HER2-targeted therapy post-biopsy. All variables were used as categorical variable: number or prior lines was divided in 4 groups, mutations and copy numbers were grouped as yes/no, and signatures were grouped in above/below median

**Fig. 2:**
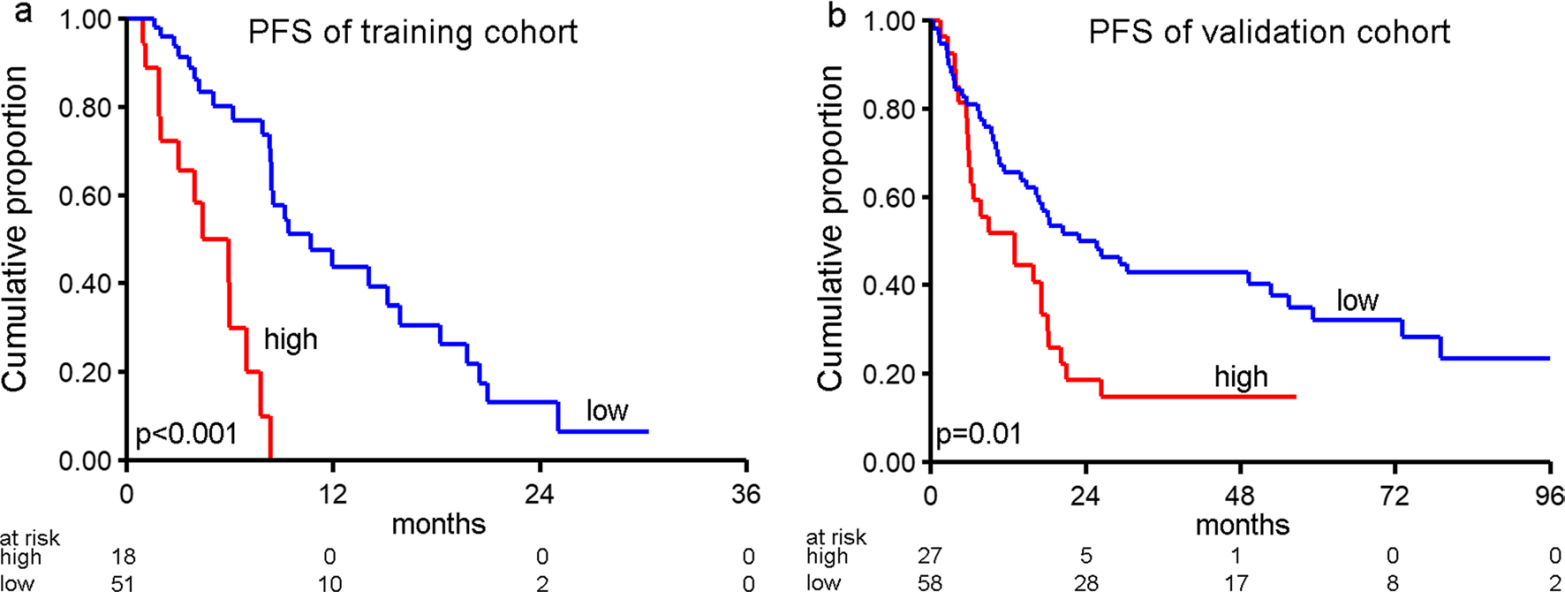
Multivariable model prediction of PFS. Kaplan–Meier progression-free survival curves of patients grouped by risk score. **a** Patients from our own cohort, labeling samples with a risk score ≥ 2.25 as high risk (see “Methods”) using five features: the number of lines of prior treatment, mutations of PIK3CA or CDK12, gain of chromosome 8p11.23 and a high contribution of gene signature DBS3. **b** Validation cohort, using PIK3CA, CDK12 mutation and Amp-peak 6 gain in the model as the only available features for the validation samples. Since these 3 features were mutually exclusive in this cohort, if any events of these 3 features were present the sample was assigned as high risk, the sample was assigned as high risk. *p* values derived from log-rank test

A single risk score was calculated by combining the five items in the model (see “Methods” section), and the optimal cut point of this score was determined to classify patients as high or low risk. These groups were significantly associated with PFS (log rank *p* < 0.001) on anti-HER2 therapy (Fig. 2a).

To validate these findings, publicly available data [11] documenting response to anti-HER2 therapy were used. From this validation cohort, we selected all metastatic breast cancer samples, which were sequenced by the MSK-IMPACT panel, covering 341 cancer-associated genes. Unfortunately, because of this targeted sequencing, DBS3 could not be reliably estimated, and furthermore, the number of prior therapy lines was unknown. However, the remaining three items (PIK3CA and CDK12 mutation status, and gain of 8p11.23) could still be combined (Fig. 2b) and again showed a clear association with PFS, also when we applied this score of the three parameters to our own cohort (log-rank test *p* < 0.001).

### Establishing a HER2-driven expression profile

**Fig. 3:**
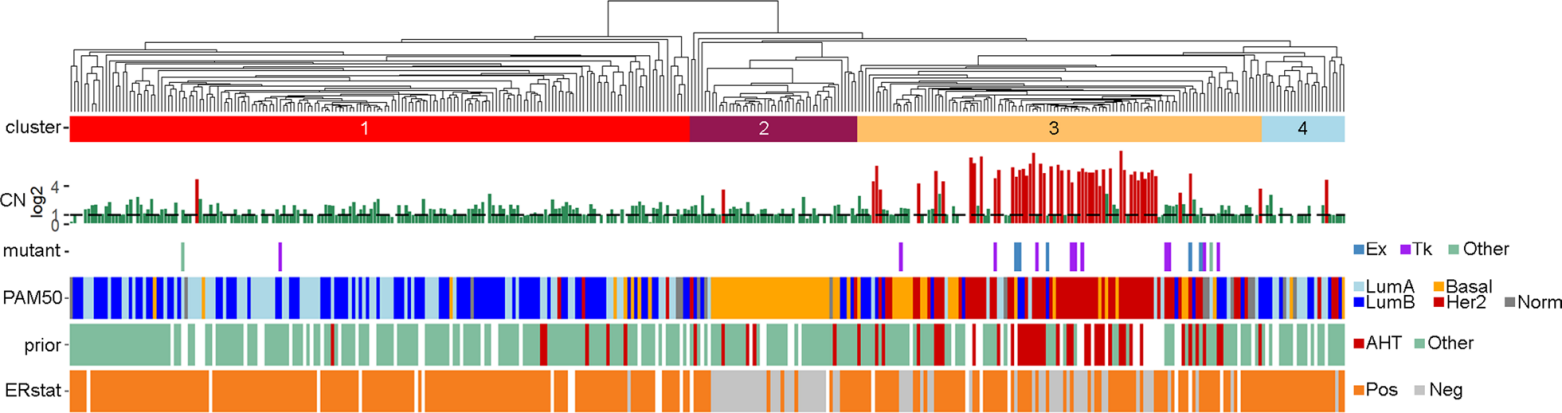
Integrative analysis of hierarchical clustering using HER2-associated genes. A gene expression-based correlation matrix was used to cluster 366 MBC cases (see Additional file 1: Figure S3 for the accompanying heatmap). Relationships with *ERBB2* log2-CN (dashed line indicates CN of 2, red indicate samples labeled as HER2+ in our analysis), *ERBB2* mutation (amino acid changing events in: Ex, Extracellular domain, Tk, Tyrosine Kinase domain), PAM50 molecular subtype estimation, anti-HER2 therapy (AHT) given prior to biopsy and the ER status are shown

For a subset of 366 MBC samples (of which 49 HER2+), RNA sequencing was performed. To further investigate differences between HER2+ cases, a HER2-driven expression profile of 878 genes was established, which were identified by comparing HER2+ versus HER2− cases (see “Methods”, Additional file 1: Table S2). Of note, expression of *ERBB2* itself and other chromosome 17 genes (which are potentially co-amplified with *ERBB2*) were excluded from this gene list. Gene expression values were used to create a correlation matrix of sample versus sample which was subsequently used for hierarchical clustering (Fig. 3, Additional file 1: Figure S3). The first (red) and last (blue) of the four cluster groups consisted of mainly ER+- and HER2-negative samples. The majority of the samples in cluster 2 (magenta) were ER negative, HER2-negative samples. Cluster 3 (yellow) was significantly enriched for HER2+ samples (47 out of 49 HER2+ samples, Fisher exact *p* = 1.94e−22) and prior anti-HER2 therapy treated cases (38 out of 49, Fisher exact *p* = 2.97e−12). The enrichment of HER2+ cases in cluster 3 was also evident when considering the PAM50 derived molecular subtypes [26] (62 out of 70 PAM50 HER2 subtypes are in cluster 3, Chi-square *p* = 8.24e−56). Remarkably, 16 of the 18 mutated *ERBB2* samples in this dataset were also in cluster 3 (Additional file 1: Table S3, Fisher exact *p* = 1.17e−5) even though 10 of these 16 were HER2− (CN below threshold).

To verify that the 878 genes used for the clustering indeed represent a HER2-driven expression profile, independent datasets of publicly available expression data of PBCs (*n* = 867) were used. Samples were similarly clustered using the HER2-specific genes and the three main clusters were associated with the PAM50 molecular subtypes and *ERBB2* expression. This again showed a clear association of the HER2-driven expression profile and HER2+ samples (i.e. *ERBB2* expression was significantly higher in the HER2-enriched cluster containing 102 out of the 112 HER2 molecular subtypes (Additional file 1: Figure S4a–c). In addition, the HER2-specific genes were analyzed using IPA (Ingenuity Pathway Analysis), focusing on the ‘upstream regulators’ part, as defined by IPA. This showed that among the genes in the HER2-driven expression profile, several overlapped with target genes of known kinases, growth- and transcription factors, the top being *ERBB2* (*p* = 2.65e−5, activation z-score 2.339) with 63 target genes in the HER2-specific gene list (see Additional file 1: Table S4 for activated regulators with *p* < 0.001). Other notable significantly activated upstream regulators are *NRG1* and *EGF*, both ligands for the ERBB family of genes, and *RAF1*, involved in the MAP kinase pathway.

### Underlying biological processes of the HER2-driven expression profile

**Fig. 4:**
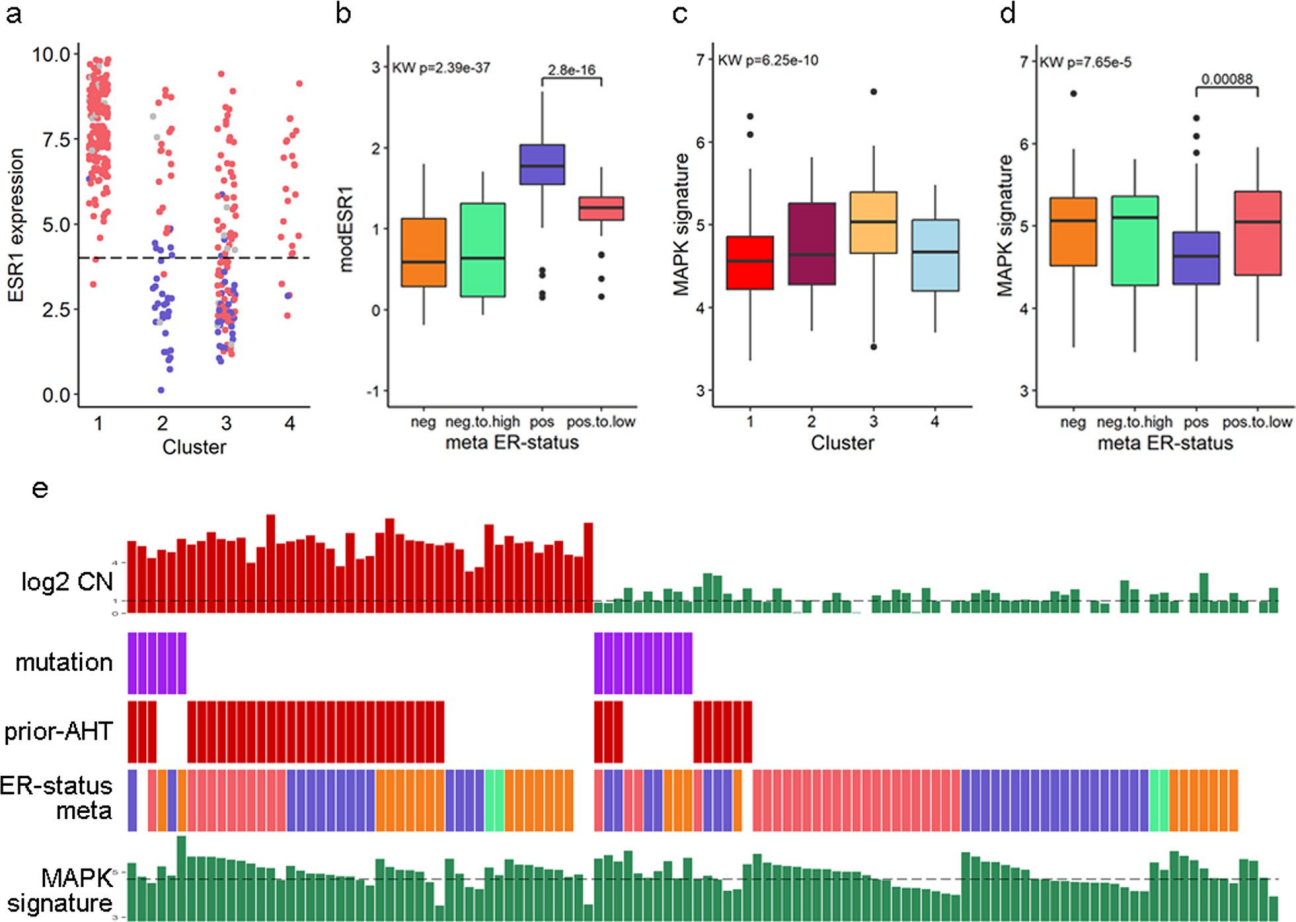
Overview of cluster 3 samples, showing a shift toward increased MAPK pathway expression. **a** *ESR1* expression in the 4 clusters, red indicates samples labeled as ER+ in their primary disease, blue ER negative in primary disease. **b** The ESR1 module score over samples, grouped by the meta ER status: a combination of MBC *ESR1* expression and reported ER status in primary (i.e. ‘pos.to.low’ indicate samples with a reported ER+ status in primary but with low *ESR1* expression in the metastatic lesion and vice versa for ‘neg.to.pos’). **c**, **d** MAPK signature expression over cluster groups (**c**) and meta ER status (**d**). **e** Summary of characteristics of cluster 3 samples. Top 2 tracks show log2 CopyNumber (dotted line indicates CN of 2) and mutation of ERBB2. The third track indicates patients who received anti-HER2 therapy prior to biopsy. Track 4 shows ER status: brown indicate cases of which the primary tumor was ER positive but the metastatic biopsy has low *ESR1* expression, i.e. ER pos-to-low. Green: ER neg-to-high, orange: concurrent ER pos, gray: concurrent ER neg. Bottom track shows expression levels of the MAPK signature, and dotted line indicates median

Combining these results, the 116 samples from cluster 3 (Fig. 3) are clearly driven by common pathways which are specific for, but not exclusively linked to, HER2−positivity since *ERBB2* is not always amplified or over-expressed (Additional file 1: Figure S5a). Since the original ER status was derived from pathology reports of the primary disease, *ESR1* expression of the metastatic samples was evaluated as well. Surprisingly, 42 samples in this dataset (11.5%) show a low expression of *ESR1* while the ER status was reported as positive in primary disease, and 36 out of these 42 samples are located in cluster 3 (Fig. 4a). Of these 36 samples, 25 do not show *ERBB2* amplification (median CN 2.01, 95% CI 1.90–2.62). In contrast, in the PBC BASIS cohort [24] *ESR1* expression levels corresponded with ER pathology status (Additional file 1: Figure S3b), suggesting that the observed ER pos-to-low cases in MBC may be an acquired trait. The ESR1 module score, a previously described gene signature associated with an active ER pathway [27], corroborates this change in ER. The median level of the ESR1 module was lower in cluster 3 versus cluster 1 and 4 (Additional file 1: Figure S5b). This was specifically observed in the ER pos-to-low cases than in the concordant ER+ cases and higher than in the concordant ER-negative cases (Kruskal–Wallis (KW) *p* = 2.39e−37, Fig. 4b).

Although the absolute percentage of ER+ cells of the primary tumor is unknown and may explain this observation, an attractive alternative hypothesis for these originally ER+ samples with low *ESR1* expression, is that the tumor cells may have switched from ER-dependent growth to using alternative pathways. Since the majority (86%) of these samples cluster together with samples previously exposed to anti-HER2 treatment, the MAPK pathway is of particular interest since it has been linked to both anti-HER2 therapy and endocrine resistance [11, 28]. Somatic mutations labeled as pathogenic (according to OncoKB) in either *ERBB2, EGFR, NF1, KRAS, BRAF* and *MAPK2 *were first used as biomarker for activated MAPK signaling [11]. Of the 23 cases with such a mutation, 12 were located in cluster 3: 9 *ERBB2* and 3 *EGFR*. All 6 *KRAS/BRAF*-mutated cases were located in cluster 1 (along with 3 *EGFR* and 2 *ERBB2* cases) and that cluster as a whole showed a lower MAPK expression signature (Fig. 4c). This is in line with results by Wagle et al. [27], showing a disconnection between *RAS/RAF* mutation status and MAPK pathway activity as determined by a MAPK expression signature.

Using this established MAPK signature, we found significantly higher expression in cluster 3 as a whole (KW *p* = 6.25e−10, Fig. 4c), but this was specifically observed in ER pos-to-low cases (KW *p* = 7.65e−5, Fig. 4d). A post hoc analysis showed a significantly higher MAPK signature (MWU *p* = 0.00088) in the ER pos-to-low samples compared to concordantly ER+ cases (i.e. primary ER+ status with high *ESR1* expression in the metastatic lesion). In PBC, the expression of the MAPK signature was significantly higher in both the HER2 cluster and the luminal-driven cluster, compared to the basal cluster (Additional file 1: Figure S4d, MWU *p* = 4.76e−9 and *p* = 3.20e−21). Considering results in MBC, which showed the MAPK signature most active in cluster 3 (Fig. 3d), this suggests that in MBC MAPK activity is mostly sustained in HER2-driven samples.

As summarized in Fig. 4e, in our metastatic BC cohort 116 samples show a HER2-driven expression profile. Forty-seven of these had a high *ERBB2* CN, while of the remaining 69 cases, 10 had a mutation in *ERBB2* and 9 had received prior anti-HER2 therapy (3 cases were both mutated and had received prior anti-HER2 therapy), but were progressive on this therapy. Of the 53 *ERBB2* low CN without an *ERBB2* mutation and not having received prior anti-HER2 therapy, 21 potentially switched from being ER-driven (ER+ according to status in primary) to HER2-driven disease (metastatic biopsy had low *ESR1* expression but co-clustered with HER2 cases) and a further 32 samples may be driven by MAPK (above median MAPK signature expression), with 12 samples showing both phenotypes (low *ESR1*/high MAPK).

## Discussion

In the present work, we performed an in-depth investigation into the genomic and transcriptomic characteristics of HER2-amplified MBC biopsies incorporating available clinical data on (prior) therapy and treatment response. To our knowledge, this is the first large cohort that reports specifically on WGS and RNAseq of HER2+ metastases, also including a comparison to publicly available WGS data of primary BC tumors.

In general, there were few genomic differences observed comparing (unpaired) metastatic vs primary HER2+ cases. The differences that were observed, namely a higher rate of *TP53* mutations and a higher contribution of signature DBS2, are either not subtype-specific (*TP53*) or does not appear highly affected in HER2+ MBC (DBS2) [25]. Similarly, no prominent changes were observed in the genome of patients that had been treated prior to biopsy with anti-HER2 therapy compared to treatment-naïve patients, implicating that resistance mechanisms are either diverse or driven by the therapeutic backbone of chemotherapy or endocrine therapy.

Analysis of the genomic characteristics in relationship to post-biopsy anti-HER2-targeted treatment revealed a strong predictive model, which could be partly validated in independent public data [11]. Predictors for fast progressive disease (< 9 months) were the presence of PIK3CA and CDK12 mutations, an amplified region on chromosome 8 (8p11.23 with ZNF703 as closest gene) and a high contribution of mutational signature DBS3. The impaired outcome on HER2-targeted therapy in the presence of PIK3CA mutations has been shown in preclinical and clinical literature many times before and has prompted clinical trials, targeting the PI3K pathway and HER2 simultaneously [11, 29–31]. A phase II trial with the pan PI3K inhibitor buparlisib showed a limited clinical benefit rate of 14%, accompanied by grade 3–4 toxicity in 70% of patients, and did not meet its primary target [32]. The less toxic alpha subunit-specific PI3K inhibitor alpelisib, however, has shown efficacy and tolerability in ER+- and HER2-negative MBC in the SOLAR-1 trial, especially for PIK3CA-mutated cases [33, 34]. The efficacy of alpelisib combined with anti-HER2-targeted therapy is now being investigated in PIK3CA-mutated patients in the ALPHABET trial (NCT05063786) [35]. CDK12 amplification has been linked to anti-HER2 therapy resistance before [36]. Amplification of (the region surrounding) ZNF703 was not described for HER2+ breast cancer, but has been linked to luminal B breast cancer before, which is known to be inherently aggressive [37]. Interestingly, the DBS3 signature has not been linked to breast cancer before [15]. Although in our set only 8 tumors (12%) harbored a contribution of > 10%, this was the strongest predictive factor in our multivariable model. Not much is known on the role of DBS3 in breast cancer. In gastrointestinal cancers, this signature has been linked to a hypermutator phenotype mediated by POLE mutations, which were not significantly enriched in our MBC cohort [38].

Supervised clustering using expression levels of a set of HER2-associated genes did result in a HER2-specific sample cluster. Remarkably, this cluster did not only contain virtually all HER2+ samples, but also included the majority of *ERBB2*-mutated samples and a substantial number of cases with an *ERBB2* CN below our threshold for HER2 positivity. In 36% of the *ERBB2* low CN metastatic samples, we observed a low expression of *ESR1*, even though their primary tumor was ER+ and as such, endocrine therapy was received. Further analyses of the gene expression data showed that samples from this HER2-specific cluster had significantly higher expression of genes that are related to the MAPK pathway, which was not observed in PBC. It can therefore be hypothesized that both resistance to endocrine therapy and expression or upregulation of the ERBB kinase pathways is correlated with MAPK upregulation. This observation is in concordance with the work of Razavi et al., who showed enrichment of MAPK alterations in a large cohort of endocrine-resistant HER2-negative tumors [28]. Together, our results provide a rationale for targeting the MAPK pathway in a subset of patients, resistant to endocrine therapy or to anti-HER2 therapy. Wagle et al. [27] showed that cell lines with an upregulated MAPK pathway on RNA level are sensitive to MAPK inhibition, independent of mutational status. Since targeting multiple pathways in patients is often limited by toxicity, gene expression analysis may provide a better snapshot of the most dominant tumor driving pathway, complementary to using specific mutations for matching patients with a specific treatment. Consequently, RNA sequencing may be indispensable to predict response on therapy and to help prioritize the best strategy to pursue, which is also substantiated by a recent large pan-cancer study [39, 40]. With the knowledge that the treatment landscape for breast cancer is changing rapidly [41], it will be of important clinical significance to use such integrative analyses to distinguish between resistant tumors primarily driven by ERBB2 aberrations and those driven by downstream pathways.

One of the main players in the rapidly changing treatment landscape is the highly efficacious anti-HER2-targeted antibody–drug conjugate trastuzumab–deruxtecan (T-DXd). On the basis of the DESTINY trials, this agent additionally gained approval for the HER2-low-expressing subgroup [42, 43]. However, the current clinical workflow defining HER2 status, involving IHC and/or FISH does not seem discriminative enough the define this subgroup [44, 45]. Moreover, the recently published DAISY trial even showed that although HER2 expression is the most determinant of T-DXd efficacy, objective responses could also be achieved in the HER2 ‘ultra-low’ subgroup [46]. In line with the foregoing results, we observed comparable *ERBB2* expression across the defined clusters. We did not have formal IHC results from the metastatic lesions to make a comparison, but from our exploratory analysis we have no reason to assume that the clinical HER2 low subgroup can be distinguished on genomic level.

Although this dataset is unique with respect to its size and the availability of paired WGS and RNAseq data, the pathological assessment of the HER2 status of the metastatic tissue was lacking. Therefore, the HER2+ amplification status was inferred from the genomic data. To prevent false positives, we set a stringent copy number threshold for calling a sample HER2+, potentially resulting in a number of false negative cases. We estimated the false negative rate at 5%, derived by the number of patients who were HER2− according to our copy number threshold, but who were treated with anti-HER2 therapy prior to the metastatic biopsy, suggesting a HER2+ status for the primary tumor. However, using this stringent cutoff, WGS data from 68 truly HER2+ MBC could be compared to publicly available WGS data from PBC. Another limitation was the heterogeneity in pretreatment, especially in the drugs that accompanied anti-HER2 treatment, although we did correct for this in our analyses. Next, for clinical practice, repeated biopsies are most likely not always feasible. Lastly, our cohort is large, but some of our analysis are underpowered to draw conclusions on the absence of HER2-specific aberrations at the genomic level.

## Conclusions

WGS analysis showed that (unpaired) primary and metastatic HER2-positive breast cancers were quite similar, even when anti-HER2 treatment was administered prior to a biopsied metastasis. This points to a relatively stable genome over time for HER2-positive breast cancer. However, our results do provide clues toward underlying markers that are related to fast progression on post-biopsy anti-HER2 treatment. In addition, a distinct HER2+-associated expression profile clustered not only HER2-amplified and *ERBB2*-mutated tumors together, but also tumors without HER2 alterations, showing downregulation of estrogen-dependent signaling and upregulation of MAPK signaling. Giving the potential clinical relevance of our findings, transcriptomics should be investigated alongside genomic biomarkers in BC for developing new therapeutic strategies.

### Supplementary Information

**Additional file 1:** Supplementary Figures and Tables.

## Data Availability

WGS, RNAseq and clinical data used in this study are available under restricted access (data request number DR-068). Access can be requested by contacting the Hartwig Medical Foundation (https://hartwigmedicalfoundation.nl). The downloaded data that were used for analyses comprise somatic events per sample (SNVs, SVs, CNAs, etc.) which were generated and made available by Hartwig using code available at https://github.com/hartwigmedical/. Data from the HER2+ samples in the BASIS cohort [24] were extracted from the European Genome-phenome Archive (accession code EGAS00001001178). Data from Smith et al. [11] were retrieved from the cBioPortal for Cancer Genomics (https://www.cbioportal.org/study/summary?id=brca_mapk_hp_msk_2021).

## Abbreviations

CNV: Copy number variation
ER: Estrogen receptor
HER2: Human epidermal growth factor receptor 2
MAPK: Mitogen-activated protein kinase
MBC: Metastatic breast cancer
PBC: Primary breast cancer
TMB: Tumor mutational burden
WGS: Whole-genome sequencing
