# Supplementary material for: Integrative whole-genome and transcriptome analysis of HER2-amplified metastatic breast cancer
Source: Breast Cancer Res. 2023 Nov 15;25:145. doi: 10.1186/s13058-023-01743-z (PMC10648326; doi:10.1186/s13058-023-01743-z)
Supplement: Supplementary file 1 — Additional file 1: Supplementary Figures and Tables. [file 13058_2023_1743_MOESM1_ESM.pdf]

Supplementary Figures and Tables

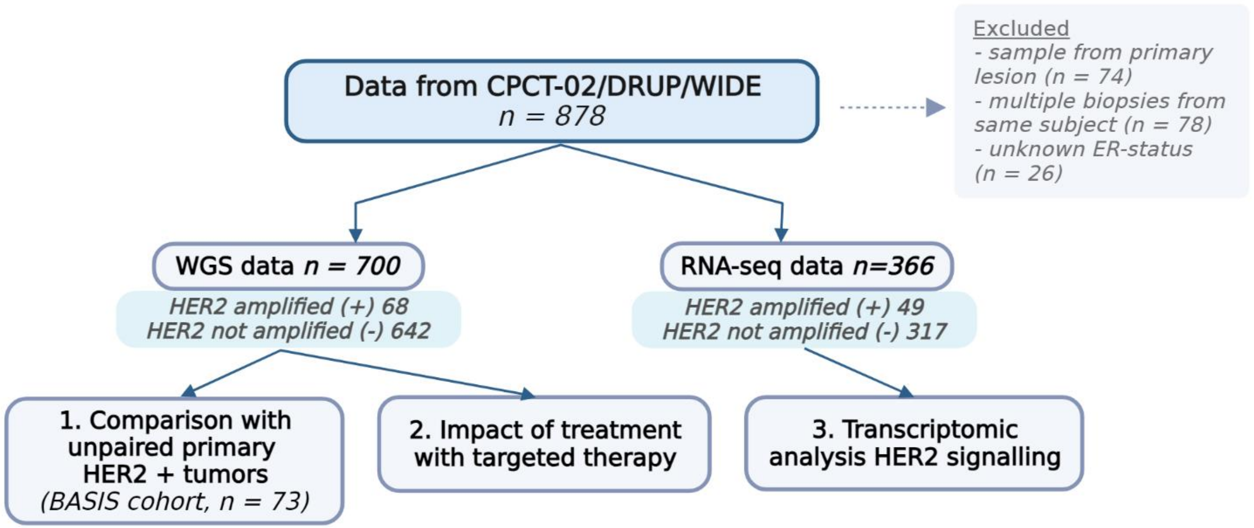

Supplementary Figure 1. Study workflow.

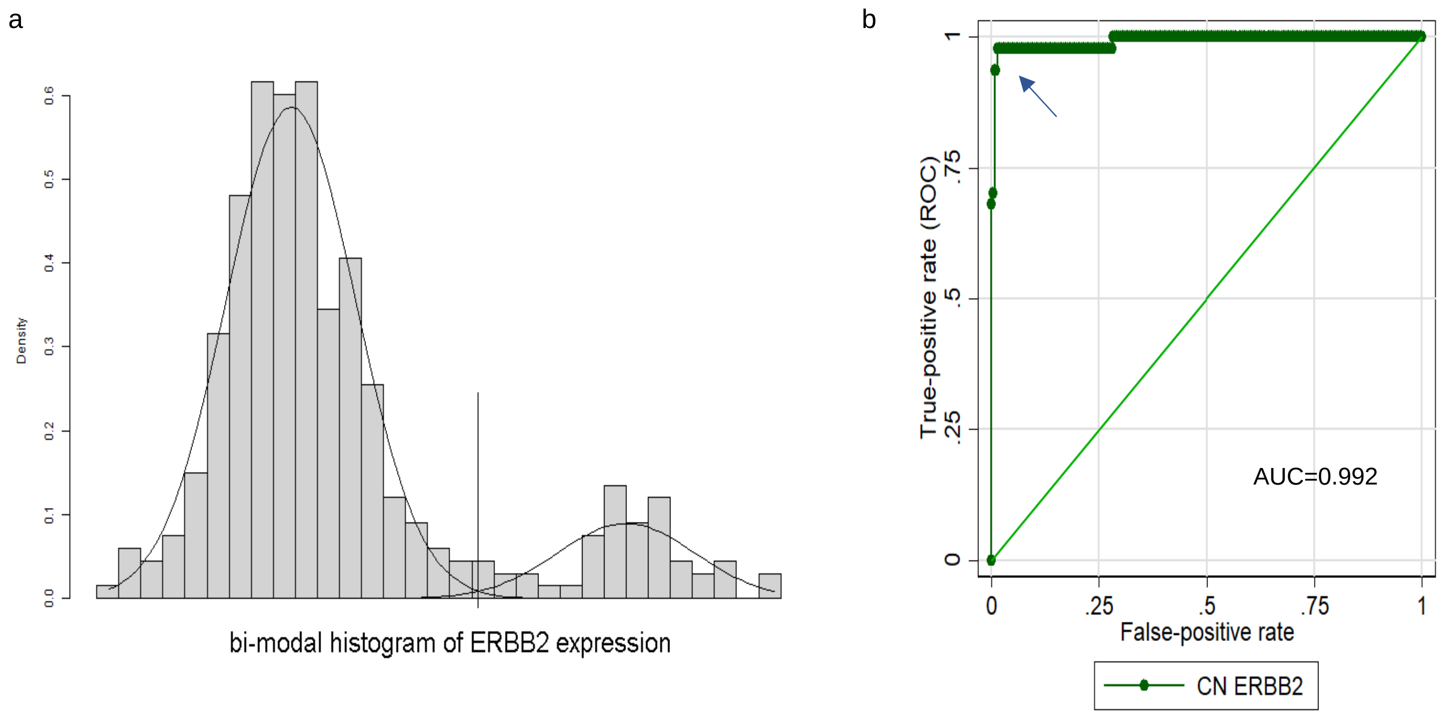

Supplementary Figure 2. ERBB2 expression and copy number threshold. a) ERBB2 expression shows a bi-modal distribution. The cross-point of the 2 normal estimation curves (vertical line) was used as threshold. b) ROC curve of ERBB2 CN, grouping samples by expression threshold. The arrow indicates the CN threshold of 9.954 (Youden index 0.965).

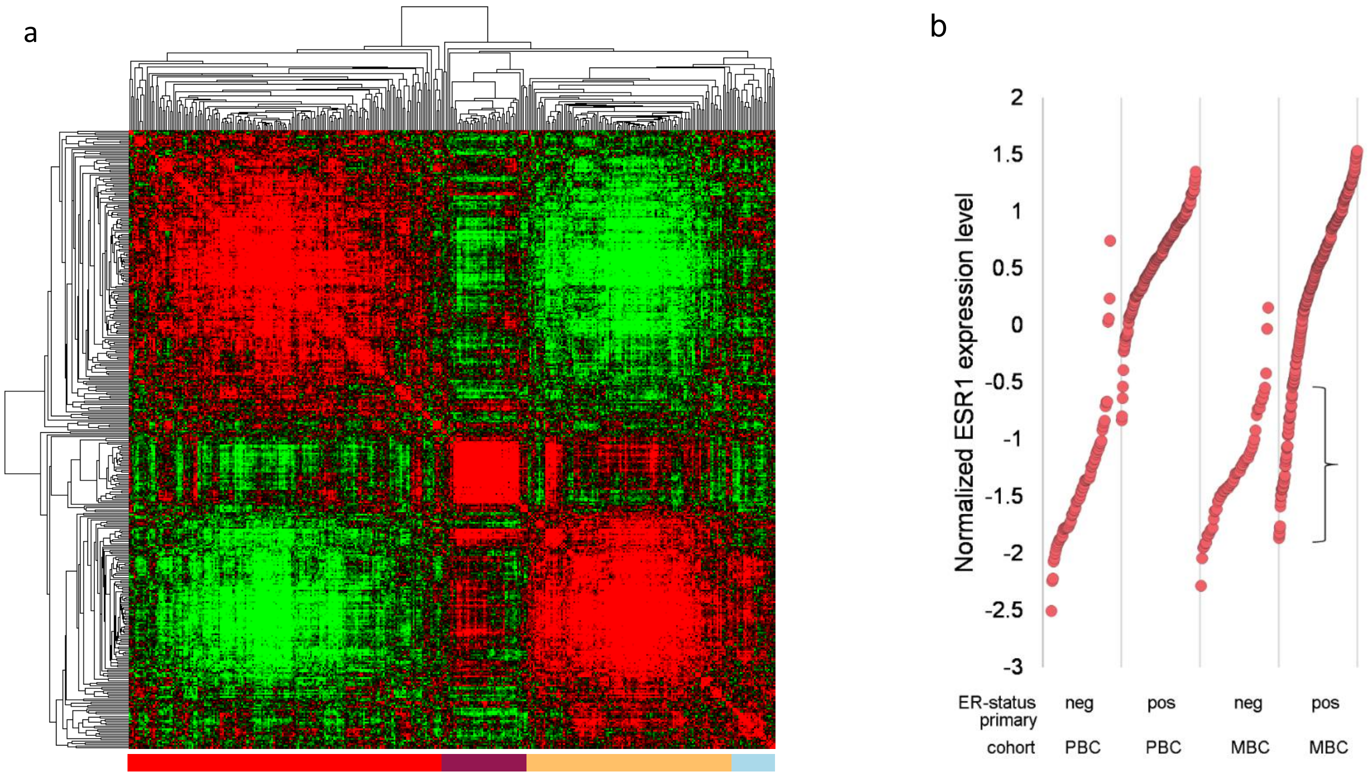

Supplementary Figure 3. Hierarchical clustering correlation matrix of RNA sequencing data of the 366 MBC samples and *ESR1* expression over cohorts. a) Hierarchical clustering correlation matrix. Expression levels of 878 genes associated with a HER2 driven expression profile were median centered and used to create a correlation matrix of sample vs sample. This matrix was subsequently used for hierarchical clustering, red and green indicating positive and negative correlation-coefficients, respectively. b) *ESR1* expression by ER-status of primary disease (via pathology reports) and BC groups. To obtain comparable levels, *ESR1* expression levels were z-normalized for PBC/MBC separately (i.e. both PBC and MBC distributions had a mean of 0 and standard deviation of 1). Bracket indicates overlapping area where ER positive cases (by status in primary disease) show expression levels comparable to ER negative cases.

a

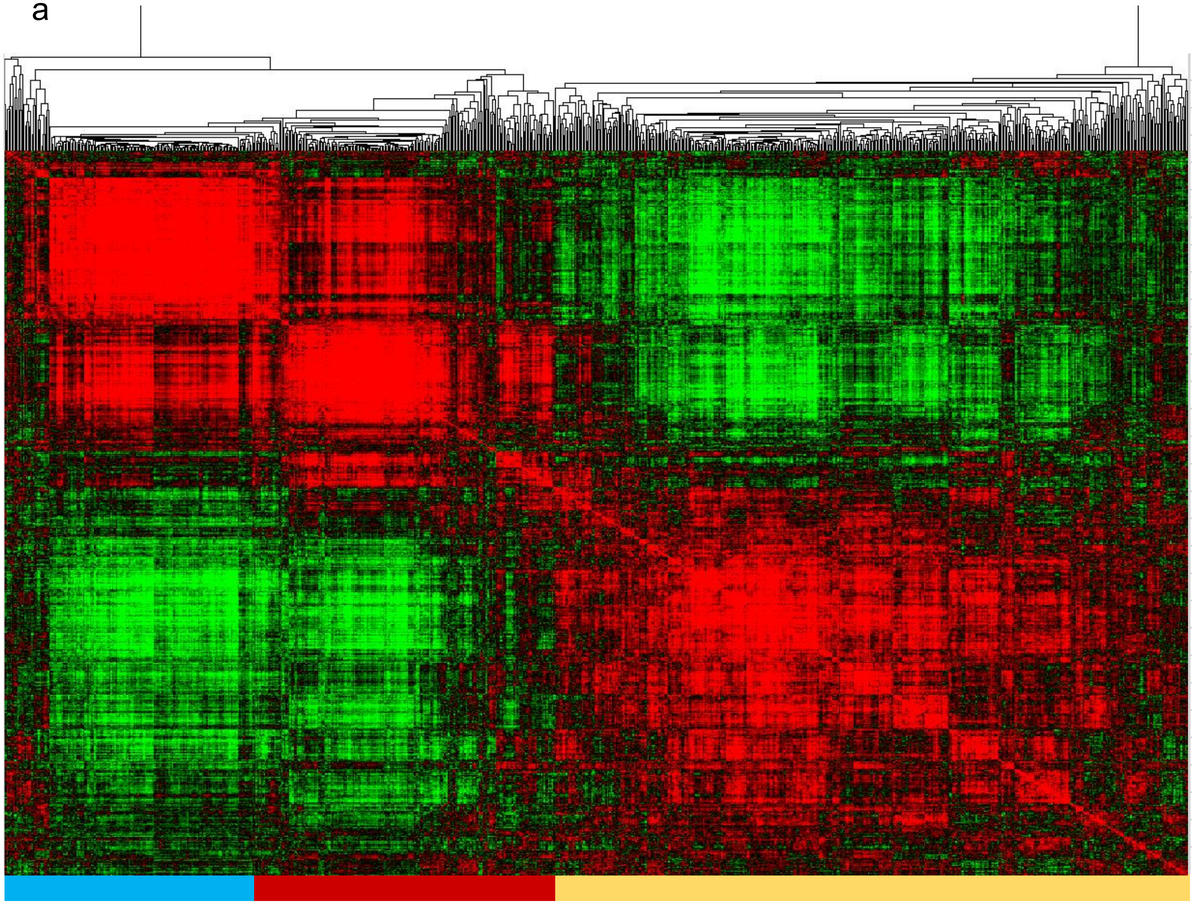

b

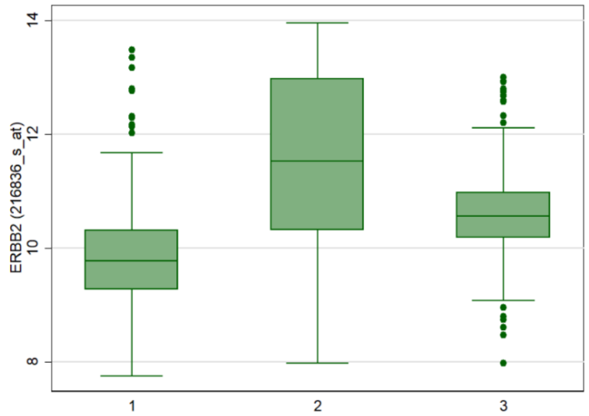

c

Subtype	cluster 1	2	3	Total
Basal	150	43	0	193
Her2	6	102	4	112
LumA	0	15	212	227
LumB	4	22	117	143
Normal	23	38	131	192
Total	183	220	464	867

d

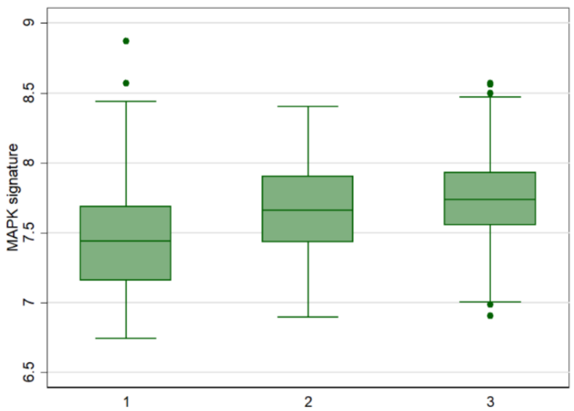

Supplementary Figure 4. Hierarchical clustering correlation matrix independent public data of PBC. a) Expression levels of 878 genes associated with a HER2 driven expression profile were median centered to create a correlation matrix of sample vs sample. This matrix was subsequently used for hierarchical clustering, red and green indicating positive and negative correlation-coefficients, respectively. b) main clusters from (a) versus ERBB2 expression levels. c) versus molecular subtypes. d) versus average expression of MAPK signature genes.

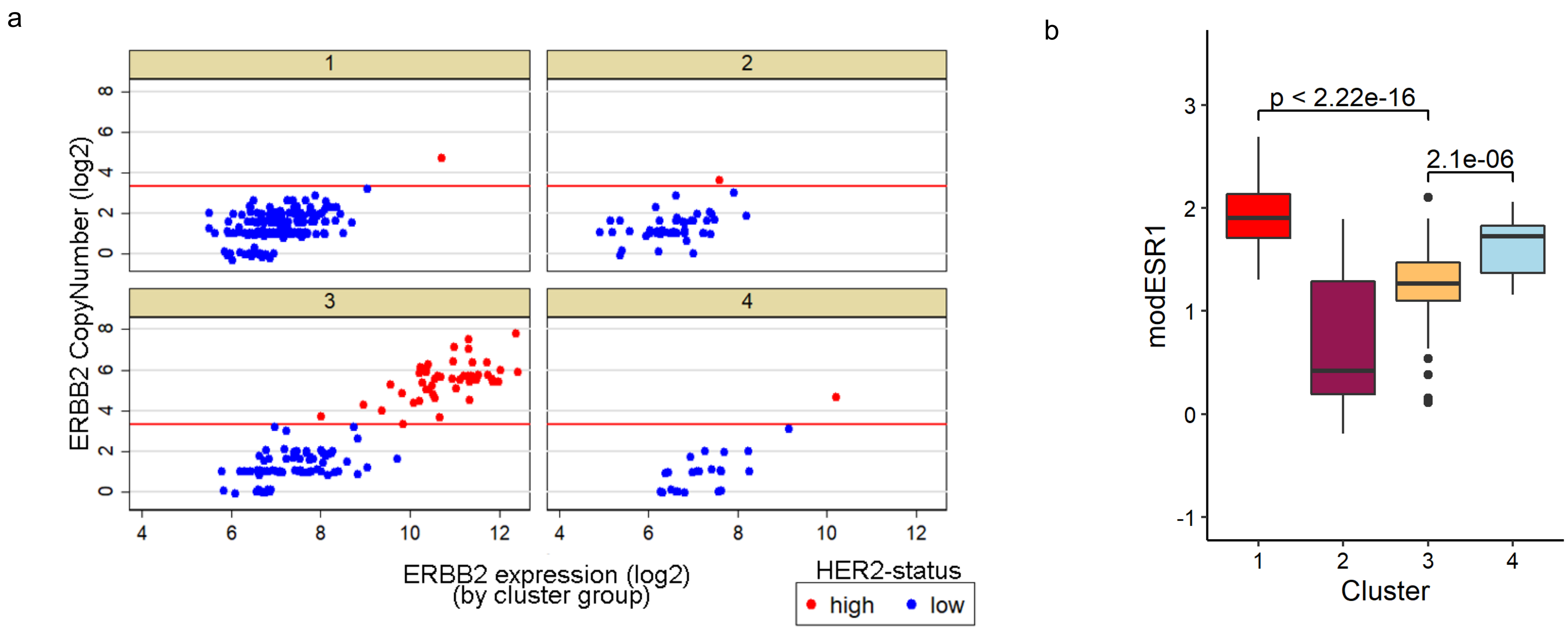

Supplementary Figure 5. ERBB2 copy number and expression by cluster group and ESR1 pathway score by cluster group. a) ERBB2 copy number (log2) and ERBB2 expression by cluster group. Red indicates HER2+ samples. b) ESR1 pathway expression by cluster group. The score on this module was significantly lower than in cluster 1 and 4, which also had a high frequency of ER-positive primary samples.

Supplementary Table 1: Univariable items tested for outcome on AHT

**categorical**	**remark**
ER-Status	ER+ as group 1
HER2+ Status	HER2+ as group 1
Nr of prior therapy lines	binary: 0 and 1 line as group 0
TMB_10_5	TMB grouped 0-5,5-10,>10
Whole Genome Duplication	yes/no
Chromotrypsis	yes/no
PIK3CA	mutation yes/no
ERBB2	mutation yes/no
domain	mutation ERBB2 by domain (Extracellular / Tyrosine Kinase Domain)
penp	combination of PIK3CA, ERBB2, NF1, PTEN
TP53	mutation yes/no
PIK3CA	mutation yes/no
MUC16	mutation yes/no
HMCN1	mutation yes/no
RYR2	mutation yes/no
USH2A	mutation yes/no
HYDIN	mutation yes/no
CDK12	mutation yes/no
OBSCN	mutation yes/no
ABCA13	mutation yes/no
CACNA1G	mutation yes/no
CSMD1	mutation yes/no
PKHD1L1	mutation yes/no
RYR1	mutation yes/no
SYNE2	mutation yes/no
ATR	mutation yes/no
CSMD3	mutation yes/no

**categorical**	**remark**
LRP1B	mutation yes/no
MED1	mutation yes/no
PLEC	mutation yes/no
VPS13B	mutation yes/no
ZFHX4	mutation yes/no
Amp1	Gistic region yes/no
Amp2	Gistic region yes/no
Amp3	Gistic region yes/no
Amp4	Gistic region yes/no
Amp5	Gistic region yes/no
Amp6	Gistic region yes/no
Amp7	Gistic region yes/no
Amp8	Gistic region yes/no
Amp9	Gistic region yes/no
Amp10	Gistic region yes/no
Amp11	Gistic region yes/no
Amp12	Gistic region yes/no
Amp13	Gistic region yes/no
Amp14	Gistic region yes/no
Amp15	Gistic region yes/no
Amp16	Gistic region yes/no
Del17	Gistic region yes/no
Del18	Gistic region yes/no
Del20	Gistic region yes/no
Del31	Gistic region yes/no

continuous	remark
Nr of prior therapy lines	
Muts.InDel	nr of somatic Insertion/Deletions
Muts.MNV	nr of somatic multinucleotide substitutions
Muts.SNV	nr of somatic singlenucleotide substitutions
TMB	
SV.DEL	nr of structural variant of type deletion
SV.DUP	nr of structural variant of type tandem duplication
SV.INV	nr of structural variant of type inversion
SV.SINGLE	nr of structural variant of type single
SV.TRA	nr of structural variant of type translocation
totalSV	total nr of SVs
genomePloidy	
SBS1	% contribution
SBS2	% contribution
SBS3	% contribution
SBS5	% contribution
SBS8	% contribution
SBS13	% contribution
SBS18	% contribution
SBS39	% contribution
SBS40	% contribution
DBS2	% contribution
DBS3	% contribution
DBS4	% contribution
DBS5	% contribution
DBS6	% contribution
DBS7	% contribution
DBS9	% contribution
DBS11	% contribution

continuous	remark
ID1	% contribution
ID2	% contribution
ID3	% contribution
ID4	% contribution
ID5	% contribution
ID6	% contribution
ID8	% contribution
ID9	% contribution
ID10	% contribution
SV2	% contribution
SV4	% contribution
SV5	% contribution
SV6	% contribution
nr_GISTIC2	Number of CNV regions via GISTIC

Supplementary Table 2: HER2-specific genes

ENSG	NAME	ENSG	NAME	ENSG	NAME
ENSG00000178372	CALML5	ENSG00000187634	SAMD11	ENSG00000117115	PADI2
ENSG00000176887	SOX11	ENSG00000229453	SPINK8	ENSG00000118514	ALDH8A1
ENSG00000198729	PPP1R14C	ENSG00000213420	GPC2	ENSG00000135472	FAIM2
ENSG00000198535	C2CD4A	ENSG00000131015	ULBP2	ENSG00000120875	DUSP4
ENSG00000172551	MUCL1	ENSG00000115850	LCT	ENSG00000159166	LAD1
ENSG00000165125	TRPV6	ENSG00000101846	STS	ENSG00000230601	TEX48
ENSG00000172478	MAB21L4	ENSG00000189377	CXCL17	ENSG00000058335	RASGRF1
ENSG00000163220	S100A9	ENSG00000102970	CCL17	ENSG00000081148	IMPG2
ENSG00000134323	MYCN	ENSG00000249961	TERB1	ENSG00000125898	FAM110A
ENSG00000143546	S100A8	ENSG00000130675	MNX1	ENSG00000258947	TUBB3
ENSG00000179913	B3GNT3	ENSG00000203985	LDLRAD1	ENSG00000101333	PLCB4
ENSG00000137648	TMPRSS4	ENSG00000182782	HCAR2	ENSG00000104941	RSPH6A
ENSG00000163362	INAVA	ENSG00000133962	CATSPERB	ENSG00000188483	IER5L
ENSG00000115221	ITGB6	ENSG00000132746	ALDH3B2	ENSG00000088305	DNMT3B
ENSG00000158089	GALNT14	ENSG00000081985	IL12RB2	ENSG00000183496	MEX3B
ENSG00000107242	PIP5K1B	ENSG00000143512	HHIPL2	ENSG00000071575	TRIB2
ENSG00000261115	TMEM178B	ENSG00000162949	CAPN13	ENSG00000006118	TMEM132A
ENSG00000105523	FAM83E	ENSG00000135698	MPHOSPH6	ENSG00000140832	MARVELD3
ENSG00000069812	HES2	ENSG00000088836	SLC4A11	ENSG00000163993	S100P
ENSG00000177202	SPACA4	ENSG00000088726	TMEM40	ENSG00000184343	SRPK3
ENSG00000166268	MYRFL	ENSG00000128165	ADM2	ENSG00000170684	ZNF296
ENSG00000127249	ATP13A4	ENSG00000099994	SUSD2	ENSG00000223573	TINCR
ENSG00000137975	CLCA2	ENSG00000148377	IDI2	ENSG00000165202	OR1Q1
ENSG00000205502	C2CD4B	ENSG00000102387	TAF7L	ENSG00000171208	NETO2
ENSG00000120471	TP53AIP1	ENSG00000135480	KRT7	ENSG00000151117	TMEM86A
ENSG00000168389	MFSD2A	ENSG00000182308	DCAF4L1	ENSG00000140798	ABCC12
ENSG00000131019	ULBP3	ENSG00000103089	FA2H	ENSG00000160867	FGFR4

ENSG	NAME	ENSG	NAME	ENSG	NAME
ENSG00000063127	SLC6A16	ENSG00000169583	CLIC3	ENSG00000121270	ABCC11
ENSG00000184709	LRRC26	ENSG00000182902	SLC25A18	ENSG00000135750	KCNK1
ENSG00000185863	TMEM210	ENSG00000131379	C3orf20	ENSG00000205426	KRT81
ENSG00000086548	CEACAM6	ENSG00000130783	CCDC62	ENSG00000106789	CORO2A
ENSG00000148215	OR5C1	ENSG00000149506	ZP1	ENSG00000181588	MEX3D
ENSG00000181649	PHLDA2	ENSG00000110400	NECTIN1	ENSG00000203485	INF2
ENSG00000188959	C9orf152	ENSG00000230054	TEX53	ENSG00000253309	SERPINE3
ENSG00000135248	FAM71F1	ENSG00000116703	PDC	ENSG00000198369	SPRED2
ENSG00000248099	INSL3	ENSG00000115339	GALNT3	ENSG00000102554	KLF5
ENSG00000106077	ABHD11	ENSG00000118557	PMFBP1	ENSG00000181016	LSMEM1
ENSG00000186193	SAPCD2	ENSG00000160211	G6PD	ENSG00000102996	MMP15
ENSG00000179057	IGSF22	ENSG00000212747	RTL8B	ENSG00000181090	EHMT1
ENSG00000181019	NQO1	ENSG00000115884	SDC1	ENSG00000171219	CDC42BPG
ENSG00000105088	OLFM2	ENSG00000003249	DBNDD1	ENSG00000131037	EPS8L1
ENSG00000187566	NHLRC1	ENSG00000241484	ARHGAP8	ENSG00000170537	TMC7
ENSG00000117009	KMO	ENSG00000103067	ESRP2	ENSG00000185674	LYG2
ENSG00000197119	SLC25A29	ENSG00000134909	ARHGAP32	ENSG00000248405	PRR5-ARHGAP8
ENSG00000100031	GGT1	ENSG00000198807	PAX9	ENSG00000147689	FAM83A
ENSG00000157613	CREB3L1	ENSG00000146386	ABRACL	ENSG00000111644	ACRBP
ENSG00000188322	SBK1	ENSG00000198753	PLXNB3	ENSG00000051620	HEBP2
ENSG00000161911	TREML1	ENSG00000167800	TBX10	ENSG00000261609	GAN
ENSG00000111981	ULBP1	ENSG00000196639	HRH1	ENSG00000144214	LYG1
ENSG00000166866	MYO1A	ENSG00000184635	ZNF93	ENSG00000188050	RNF133
ENSG00000104894	CD37	ENSG00000135269	TES	ENSG00000196781	TLE1
ENSG00000189030	VHLL	ENSG00000198363	ASPH	ENSG00000135245	HILPDA
ENSG00000078804	TP53INP2	ENSG00000197070	ARRDC1	ENSG00000139988	RDH12
ENSG00000100290	BIK	ENSG00000130164	LDLR	ENSG00000172183	ISG20
ENSG00000151715	TMEM45B	ENSG00000197506	SLC28A3	ENSG00000188643	S100A16

ENSG	NAME	ENSG	NAME	ENSG	NAME
ENSG00000112599	GUCA1B	ENSG00000197496	SLC2A10	ENSG00000149782	PLCB3
ENSG00000139318	DUSP6	ENSG00000173261	PLAC8L1	ENSG00000203950	RTL8A
ENSG00000178026	LRRC75B	ENSG00000240038	AMY2B	ENSG00000134317	GRHL1
ENSG00000069399	BCL3	ENSG00000103460	TOX3	ENSG00000064270	ATP2C2
ENSG00000272398	CD24	ENSG00000169758	TMEM266	ENSG00000101255	TRIB3
ENSG00000135643	KCNMB4	ENSG00000145555	MYO10	ENSG00000106003	LFNG
ENSG00000177025	C19orf18	ENSG00000143013	LMO4	ENSG00000184731	FAM110C
ENSG00000125257	ABCC4	ENSG00000102385	DRP2	ENSG00000135540	NHSL1
ENSG00000183421	RIPK4	ENSG00000130529	TRPM4	ENSG00000146072	TNFRSF21
ENSG00000124664	SPDEF	ENSG00000178409	BEND3	ENSG00000077044	DGKD
ENSG00000165555	NOXRED1	ENSG00000239264	TXNDC5	ENSG00000102898	NUTF2
ENSG00000184828	ZBTB7C	ENSG00000240849	PEDS1	ENSG00000075218	GTSE1
ENSG00000156966	B3GNT7	ENSG00000172780	RAB43	ENSG00000124766	SOX4
ENSG00000168528	SERINC2	ENSG00000136295	TTYH3	ENSG00000006432	MAP3K9
ENSG00000064547	LPAR2	ENSG00000114315	HES1	ENSG00000080845	DLGAP4
ENSG00000099889	ARVCF	ENSG00000106089	STX1A	ENSG00000185340	GAS2L1
ENSG00000162639	HENMT1	ENSG00000176973	FAM89B	ENSG00000125247	TMTC4
ENSG00000258429	PDF	ENSG00000164638	SLC29A4	ENSG00000100359	SGSM3
ENSG00000171786	NHLH1	ENSG00000175567	UCP2	ENSG00000119772	DNMT3A
ENSG00000196411	EPHB4	ENSG00000183963	SMTN	ENSG00000186187	ZNRF1
ENSG00000138074	SLC5A6	ENSG00000133422	MORC2	ENSG00000112242	E2F3
ENSG00000071564	TCF3	ENSG00000167508	MVD	ENSG00000104866	PPP1R37
ENSG00000143061	IGSF3	ENSG00000198911	SREBF2	ENSG00000275183	LENG9
ENSG00000164049	FBXW12	ENSG00000074416	MGLL	ENSG00000078237	TIGAR
ENSG00000183762	KREMEN1	ENSG00000113758	DBN1	ENSG00000118263	KLF7
ENSG00000102984	ZNF821	ENSG00000125895	TMEM74B	ENSG00000168140	VASN
ENSG00000196338	NLGN3	ENSG00000155714	PDZD9	ENSG00000010818	HIVEP2
ENSG00000104881	PPP1R13L	ENSG00000143507	DUSP10	ENSG00000112787	FBRSL1
ENSG00000197442	MAP3K5	ENSG00000169925	BRD3	ENSG00000051128	HOMER3

ENSG	NAME	ENSG	NAME	ENSG	NAME
ENSG00000160190	SLC37A1	ENSG00000149418	ST14	ENSG00000198792	TMEM184B
ENSG00000107130	NCS1	ENSG00000196576	PLXNB2	ENSG00000254440	PBOV1
ENSG00000130881	LRP3	ENSG00000196123	KIAA0895L	ENSG00000151883	PARP8
ENSG00000160439	RDH13	ENSG00000147144	CCDC120	ENSG00000167523	SPATA33
ENSG00000167535	CACNB3	ENSG00000011422	PLAUR	ENSG00000102349	KLF8
ENSG00000131149	GSE1	ENSG00000177169	ULK1	ENSG00000140848	CPNE2
ENSG00000112655	PTK7	ENSG00000125772	GPCPD1	ENSG00000102871	TRADD
ENSG00000173457	PPP1R14B	ENSG00000109625	CPZ	ENSG00000131061	ZNF341
ENSG00000143995	MEIS1	ENSG00000215529	EFCAB8	ENSG00000092820	EZR
ENSG00000100266	PACSIN2	ENSG00000189306	RRP7A	ENSG00000081803	CADPS2
ENSG00000204569	PPP1R10	ENSG00000119514	GALNT12	ENSG00000188290	HES4
ENSG00000140374	ETFA	ENSG00000139832	RAB20	ENSG00000100075	SLC25A1
ENSG00000188486	H2AX	ENSG00000103248	MTHFSD	ENSG00000134590	RTL8C
ENSG00000184545	DUSP8	ENSG00000165684	SNAPC4	ENSG00000093010	COMT
ENSG00000126214	KLC1	ENSG00000277443	MARCKS	ENSG00000005238	FAM214B
ENSG00000254986	DPP3	ENSG00000110047	EHD1	ENSG00000257923	CUX1
ENSG00000126458	RRAS	ENSG00000146410	MTFR2	ENSG00000238227	TMEM250
ENSG00000165389	SPTSSA	ENSG00000131165	CHMP1A	ENSG00000160796	NBEAL2
ENSG00000167470	MIDN	ENSG00000147166	ITGB1BP2	ENSG00000160325	CACFD1
ENSG00000146904	EPHA1	ENSG00000100403	ZC3H7B	ENSG00000125753	VASP
ENSG00000139722	VPS37B	ENSG00000133818	RRAS2	ENSG00000178386	ZNF223
ENSG00000160883	HK3	ENSG00000102390	PBDC1	ENSG00000125834	STK35
ENSG00000175274	TP53I11	ENSG00000135723	FHOD1	ENSG00000167657	DAPK3
ENSG00000148396	SEC16A	ENSG00000133275	CSNK1G2	ENSG00000163872	YEATS2
ENSG00000165724	ZMYND19	ENSG00000130669	PAK4	ENSG00000097007	ABL1
ENSG00000196526	AFAP1	ENSG00000114423	CBLB	ENSG00000102804	TSC22D1
ENSG00000136830	NIBAN2	ENSG00000205078	SYCE1L	ENSG00000175220	ARHGAP1
ENSG00000106799	TGFBR1	ENSG00000095383	TBC1D2	ENSG00000159840	ZYX
ENSG00000187678	SPRY4	ENSG00000103005	USB1	ENSG00000075240	GRAMD4

ENSG	NAME	ENSG	NAME	ENSG	NAME
ENSG00000173153	ESRRA	ENSG00000160305	DIP2A	ENSG00000099814	CEP170B
ENSG00000099956	SMARCB1	ENSG00000095951	HIVEP1	ENSG00000065457	ADAT1
ENSG00000197081	IGF2R	ENSG00000183255	PTTG1IP	ENSG00000088038	CNOT3
ENSG00000130787	HIP1R	ENSG00000008083	JARID2	ENSG00000137166	FOXP4
ENSG00000110697	PITPNM1	ENSG00000170340	B3GNT2	ENSG00000090372	STRN4
ENSG00000072274	TFRC	ENSG00000128298	BAIAP2L2	ENSG00000119139	TJP2
ENSG00000041982	TNC	ENSG00000067955	CBFB	ENSG00000160285	LSS
ENSG00000188818	ZDHHC11	ENSG00000160877	NACC1	ENSG00000130347	RTN4IP1
ENSG00000185467	KPNA7	ENSG00000103335	PIEZO1	ENSG00000184792	OSBP2
ENSG00000188566	NDOR1	ENSG00000089159	PXN	ENSG00000100156	SLC16A8
ENSG00000162645	GBP2	ENSG00000271303	SRXN1	ENSG00000101294	HM13
ENSG00000182218	HHIPL1	ENSG00000167173	C15orf39	ENSG00000123130	ACOT9
ENSG00000034510	TMSB10	ENSG00000158470	B4GALT5	ENSG00000135048	CEMIP2
ENSG00000112137	PHACTR1	ENSG00000160271	RALGDS	ENSG00000205336	ADGRG1
ENSG00000100401	RANGAP1	ENSG00000241962	RP11-111H13.1	ENSG00000164054	SHISA5
ENSG00000015475	BID	ENSG00000173214	MFSD4B	ENSG00000130706	ADRM1
ENSG00000159399	HK2	ENSG00000198373	WWP2	ENSG00000103111	MON1B
ENSG00000070010	UFD1	ENSG00000074181	NOTCH3	ENSG00000203883	SOX18
ENSG00000006459	KDM7A	ENSG00000167378	IRGQ	ENSG00000186716	BCR
ENSG00000072401	UBE2D1	ENSG00000197136	PCNX3	ENSG00000129566	TEP1
ENSG00000119682	AREL1	ENSG00000106683	LIMK1	ENSG00000141985	SH3GL1
ENSG00000141959	PFKL	ENSG00000136378	ADAMTS7	ENSG00000118960	HS1BP3
ENSG00000103042	SLC38A7	ENSG00000104142	VPS18	ENSG00000198752	CDC42BPB
ENSG00000089639	GMIP	ENSG00000160310	PRMT2	ENSG00000171443	ZNF524
ENSG00000145979	TBC1D7	ENSG00000137094	DNAJB5	ENSG00000139209	SLC38A4
ENSG00000176619	LMNB2	ENSG00000278662	GOLGA6L10	ENSG00000196588	MRTFA
ENSG00000114554	PLXNA1	ENSG00000164047	CAMP	ENSG00000116871	MAP7D1
ENSG00000106348	IMPDH1	ENSG00000105281	SLC1A5	ENSG00000146535	GNA12

ENSG	NAME	ENSG	NAME	ENSG	NAME
ENSG00000157593	SLC35B2	ENSG00000215193	PEX26	ENSG00000042753	AP2S1
ENSG00000152558	TMEM123	ENSG00000136026	CKAP4	ENSG00000165689	ENTR1
ENSG00000105063	PPP6R1	ENSG00000174177	CTU2	ENSG00000099917	MED15
ENSG00000175463	TBC1D10C	ENSG00000124788	ATXN1	ENSG00000106868	SUSD1
ENSG00000163812	ZDHHC3	ENSG00000123989	CHPF	ENSG00000241553	ARPC4
ENSG00000188157	AGRN	ENSG00000163170	BOLA3	ENSG00000164880	INTS1
ENSG00000134882	UBAC2	ENSG00000135636	DYSF	ENSG00000120254	MTHFD1L
ENSG00000139793	MBNL2	ENSG00000007866	TEAD3	ENSG00000182944	EWSR1
ENSG00000187605	TET3	ENSG00000132970	WASF3	ENSG00000139908	TSSK4
ENSG00000050820	BCAR1	ENSG00000115216	NRBP1	ENSG00000221823	PPP3R1
ENSG00000119408	NEK6	ENSG00000198113	TOR4A	ENSG00000205903	ZNF316
ENSG00000172757	CFL1	ENSG00000104853	CLPTM1	ENSG00000147697	GSDMC
ENSG00000242802	AP5Z1	ENSG00000188986	NELFB	ENSG00000128272	ATF4
ENSG00000159720	ATP6V0D1	ENSG00000198324	PHETA1	ENSG00000148335	NTMT1
ENSG00000138835	RGS3	ENSG00000128283	CDC42EP1	ENSG00000007080	CCDC124
ENSG00000149743	TRPT1	ENSG00000075539	FRYL	ENSG00000138316	ADAMTS14
ENSG00000205250	E2F4	ENSG00000160999	SH2B2	ENSG00000130749	ZC3H4
ENSG00000101084	RAB5IF	ENSG00000181830	SLC35C1	ENSG00000137267	TUBB2A
ENSG00000103035	PSMD7	ENSG00000125970	RALY	ENSG00000137266	SLC22A23
ENSG00000142208	AKT1	ENSG00000130559	CAMSAP1	ENSG00000067829	IDH3G
ENSG00000103043	VAC14	ENSG00000105287	PRKD2	ENSG00000135047	CTSL
ENSG00000179820	MYADM	ENSG00000179055	OR13D1	ENSG00000028528	SNX1
ENSG00000135587	SMPD2	ENSG00000198517	MAFK	ENSG00000173436	MICOS10
ENSG00000142409	ZNF787	ENSG00000158526	TSR2	ENSG00000213614	HEXA
ENSG00000160218	TRAPPC10	ENSG00000087086	FTL	ENSG00000166295	ANAPC16
ENSG00000167491	GATAD2A	ENSG00000143867	OSR1	ENSG00000138303	ASCC1
ENSG00000148334	PTGES2	ENSG00000174946	GPR171	ENSG00000139624	CERS5
ENSG00000107438	PDLIM1	ENSG00000172575	RASGRP1	ENSG00000126262	FFAR2
ENSG00000182154	MRPL41	ENSG00000186496	ZNF396	ENSG00000245680	ZNF585B

ENSG	NAME	ENSG	NAME	ENSG	NAME
ENSG00000100243	CYB5R3	ENSG00000135164	DMTF1	ENSG00000184677	ZBTB40
ENSG00000180879	SSR4	ENSG00000232593	KANTR	ENSG00000065526	SPEN
ENSG00000110218	PANX1	ENSG00000007001	UPP2	ENSG00000175536	LIPT2
ENSG00000158859	ADAMTS4	ENSG00000032219	ARID4A	ENSG00000172888	ZNF621
ENSG00000172216	CEBPB	ENSG00000284773	RP11-244H3.5	ENSG00000090905	TNRC6A
ENSG00000106268	NUDT1	ENSG00000035928	RFC1	ENSG00000111785	RIC8B
ENSG00000101199	ARFGAP1	ENSG00000096717	SIRT1	ENSG00000167216	KATNAL2
ENSG00000130309	COLGALT1	ENSG00000068394	GPKOW	ENSG00000168297	PXK
ENSG00000080189	SLC35C2	ENSG00000121454	LHX4	ENSG00000183549	ACSM5
ENSG00000018280	SLC11A1	ENSG00000197841	ZNF181	ENSG00000128881	TTBK2
ENSG00000262919	CCNQ	ENSG00000109680	TBC1D19	ENSG00000139651	ZNF740
ENSG00000164818	DNAAF5	ENSG00000163257	DCAF16	ENSG00000181873	IBA57
ENSG00000214160	ALG3	ENSG00000077684	JADE1	ENSG00000113593	PPWD1
ENSG00000127914	AKAP9	ENSG00000242950	ERVW-1	ENSG00000139597	N4BP2L1
ENSG00000164535	DAGLB	ENSG00000150477	KIAA1328	ENSG00000138380	CARF
ENSG00000099256	PRTFDC1	ENSG00000176697	BDNF	ENSG00000204130	RUFY2
ENSG00000165271	NOL6	ENSG00000219626	FAM228B	ENSG00000037637	FBXO42
ENSG00000185825	BCAP31	ENSG00000165409	TSHR	ENSG00000157259	GATAD1
ENSG00000163931	TKT	ENSG00000197451	HNRNPAB	ENSG00000180011	ZADH2
ENSG00000196865	NHLRC2	ENSG00000180481	GLIPR1L2	ENSG00000165879	FRAT1
ENSG00000075624	ACTB	ENSG00000143158	MPC2	ENSG00000166275	BORCS7
ENSG00000142669	SH3BGRL3	ENSG00000123268	ATF1	ENSG00000151687	ANKAR
ENSG00000106263	EIF3B	ENSG00000145675	PIK3R1	ENSG00000122299	ZC3H7A
ENSG00000146833	TRIM4	ENSG00000204947	ZNF425	ENSG00000188542	DUSP28
ENSG00000161011	SQSTM1	ENSG00000148719	DNAJB12	ENSG00000139899	CBLN3
ENSG00000204843	DCTN1	ENSG00000131503	ANKHD1	ENSG00000089847	ANKRD24
ENSG00000095574	IKZF5	ENSG00000157873	TNFRSF14	ENSG00000165923	AGBL2
ENSG00000269858	EGLN2	ENSG00000230124	ACBD6	ENSG00000217930	PAM16

ENSG	NAME	ENSG	NAME	ENSG	NAME
ENSG00000166377	ATP9B	ENSG00000165102	HGSNAT	ENSG00000165868	HSPA12A
ENSG00000176055	MBLAC2	ENSG00000118298	CA14	ENSG00000164331	ANKRA2
ENSG00000116539	ASH1L	ENSG00000107672	NSMCE4A	ENSG00000081059	TCF7
ENSG00000050426	LETMD1	ENSG00000153237	CCDC148	ENSG00000120333	MRPS14
ENSG00000214413	BBIP1	ENSG00000165406	MARCHF8	ENSG00000197343	ZNF655
ENSG00000110888	CAPRIN2	ENSG00000159023	EPB41	ENSG00000121851	POLR3GL
ENSG00000106804	C5	ENSG00000094880	CDC23	ENSG00000168944	CEP120
ENSG00000178498	DTX3	ENSG00000179981	TSHZ1	ENSG00000108021	TASOR2
ENSG00000118579	MED28	ENSG00000095539	SEMA4G	ENSG00000170915	PAQR8
ENSG00000019995	ZRANB1	ENSG00000164296	TIGD6	ENSG00000141646	SMAD4
ENSG00000156521	TYSND1	ENSG00000170445	HARS1	ENSG00000133121	STARD13
ENSG00000134480	CCNH	ENSG00000055147	FAM114A2	ENSG00000105948	TTC26
ENSG00000188916	INSYN2A	ENSG00000145833	DDX46	ENSG00000105429	MEGF8
ENSG00000145868	FBXO38	ENSG00000151276	MAGI1	ENSG00000147548	NSD3
ENSG00000171817	ZNF540	ENSG00000132196	HSD17B7	ENSG00000175395	ZNF25
ENSG00000152217	SETBP1	ENSG00000121897	LIAS	ENSG00000113621	TXNDC15
ENSG00000126091	ST3GAL3	ENSG00000153922	CHD1	ENSG00000198961	PJA2
ENSG00000143951	WDPCP	ENSG00000139714	MORN3	ENSG00000090686	USP48
ENSG00000047188	YTHDC2	ENSG00000163001	CFAP36	ENSG00000113595	TRIM23
ENSG00000132275	RRP8	ENSG00000101353	MROH8	ENSG00000143515	ATP8B2
ENSG00000107829	FBXW4	ENSG00000152240	HAUS1	ENSG00000162623	TYW3
ENSG00000184083	FAM120C	ENSG00000188177	ZC3H6	ENSG00000033011	ALG1
ENSG00000185129	PURA	ENSG00000099290	WASHC2A	ENSG00000157796	WDR19
ENSG00000135314	KHDC1	ENSG00000155016	CYP2U1	ENSG00000215912	TTC34
ENSG00000089280	FUS	ENSG00000109790	KLHL5	ENSG00000120733	KDM3B
ENSG00000156398	SFXN2	ENSG00000135469	COQ10A	ENSG00000103510	KAT8
ENSG00000111269	CREBL2	ENSG00000143337	TOR1AIP1	ENSG00000168778	TCTN2
ENSG00000163935	SFMBT1	ENSG00000163596	ICA1L	ENSG00000204172	AGAP9
ENSG00000158987	RAPGEF6	ENSG00000143994	ABHD1	ENSG00000086589	RBM22

ENSG	NAME	ENSG	NAME	ENSG	NAME
ENSG00000170234	PWWP2A	ENSG00000121486	TRMT1L	ENSG00000138175	ARL3
ENSG00000103351	CLUAP1	ENSG00000174944	P2RY14	ENSG00000115318	LOXL3
ENSG00000134759	ELP2	ENSG00000107018	RLN1	ENSG00000188672	RHCE
ENSG00000106479	ZNF862	ENSG00000184271	POU6F1	ENSG00000172197	MBOAT1
ENSG00000162444	RBP7	ENSG00000103174	NAGPA	ENSG00000105227	PRX
ENSG00000186687	LYRM7	ENSG00000141642	ELAC1	ENSG00000168765	GSTM4
ENSG00000118777	ABCG2	ENSG00000120049	KCNIP2	ENSG00000158062	UBXN11
ENSG00000146007	ZMAT2	ENSG00000142082	SIRT3	ENSG00000120915	EPHX2
ENSG00000135622	SEMA4F	ENSG00000113108	APBB3	ENSG00000063854	HAGH
ENSG00000081791	DELE1	ENSG00000001461	NIPAL3	ENSG00000258818	RNASE4
ENSG00000103544	VPS35L	ENSG00000237190	CDKN2AIPNL	ENSG00000169313	P2RY12
ENSG00000157343	ARMC12	ENSG00000164615	CAMLG	ENSG00000176723	ZNF843
ENSG00000176783	RUFY1	ENSG00000144893	MED12L	ENSG00000167972	ABCA3
ENSG00000075407	ZNF37A	ENSG00000181631	P2RY13	ENSG00000109758	HGFAC
ENSG00000166436	TRIM66	ENSG00000196632	WNK3	ENSG00000187240	DYNC2H1
ENSG00000166313	APBB1	ENSG00000170430	MGMT	ENSG00000049769	PPP1R3F
ENSG00000113318	MSH3	ENSG00000104205	SGK3	ENSG00000111666	CHPT1
ENSG00000044459	CNTLN	ENSG00000059588	TARBP1	ENSG00000285868	CTB-109A12.2
ENSG00000185875	THNSL1	ENSG00000170099	SERPINA6	ENSG00000150593	PDCD4
ENSG00000041880	PARP3	ENSG00000154309	DISP1	ENSG00000152086	TUBA3E
ENSG00000122008	POLK	ENSG00000163749	CCDC158	ENSG00000134352	IL6ST
ENSG00000132570	PCBD2	ENSG00000131507	NDFIP1	ENSG00000112874	NUDT12
ENSG00000101773	RBBP8	ENSG00000144040	SFXN5	ENSG00000036448	MYOM2
ENSG00000182700	IGIP	ENSG00000174885	NLRP6	ENSG00000145743	FBXL17
ENSG00000096872	IFT74	ENSG00000139351	SYCP3	ENSG00000070081	NUCB2
ENSG00000204219	TCEA3	ENSG00000118276	B4GALT6	ENSG00000171160	MORN4
ENSG00000109618	SEPSECS	ENSG00000113141	IK	ENSG00000155850	SLC26A2
ENSG00000197776	KLHDC1	ENSG00000043143	JADE2	ENSG00000173258	ZNF483
ENSG00000131437	KIF3A	ENSG00000121753	ADGRB2	ENSG00000171174	RBKS

ENSG	NAME	ENSG	NAME	ENSG	NAME
ENSG00000174788	PCP2	ENSG00000129028	THAP10	ENSG00000172671	ZFAND4
ENSG00000145476	CYP4V2	ENSG00000140093	SERPINA10	ENSG00000273291	RP11-136C24.3
ENSG00000237489	C10orf143	ENSG00000145375	SPATA5	ENSG00000197375	SLC22A5
ENSG00000172661	WASHC2C	ENSG00000204314	PRRT1	ENSG00000123066	MED13L
ENSG00000133706	LARS1	ENSG00000136059	VILL	ENSG00000285043	CTD-2515O10.6
ENSG00000103540	CCP110	ENSG00000183066	WBP2NL	ENSG00000149050	ZNF214
ENSG00000115325	DOK1	ENSG00000163491	NEK10	ENSG00000163040	CCDC74A
ENSG00000144649	GASK1A	ENSG00000140443	IGF1R	ENSG00000082175	PGR
ENSG00000105707	HPN	ENSG00000132563	REEP2	ENSG00000159208	CIART
ENSG00000159307	SCUBE1	ENSG00000159374	M1AP	ENSG00000115112	TFCP2L1
ENSG00000196177	ACADSB	ENSG00000269190	FBXO17	ENSG00000151892	GFRA1
ENSG00000157303	SUSD3	ENSG00000143375	CGN	ENSG00000171791	BCL2
ENSG00000145882	PCYOX1L	ENSG00000158813	EDA	ENSG00000185739	SRL
ENSG00000150627	WDR17	ENSG00000204116	CHIC1	ENSG00000116031	CD207
ENSG00000164241	C5orf63	ENSG00000102043	MTMR8	ENSG00000168329	CX3CR1
ENSG00000158301	GPRASP2	ENSG00000179902	C1orf194	ENSG00000165953	SERPINA12
ENSG00000137821	LRRC49	ENSG00000159445	THEM4	ENSG00000160460	SPTBN4
ENSG00000171385	KCND3	ENSG00000120729	MYOT	ENSG00000171722	SPATA46
ENSG00000239887	C1orf226	ENSG00000244274	DBNDD2	ENSG00000183833	CFAP91
ENSG00000241935	HOGA1	ENSG00000167371	PRRT2	ENSG00000101098	RIMS4
ENSG00000055163	CYFIP2	ENSG00000164742	ADCY1	ENSG00000240204	SMKR1
ENSG00000010438	PRSS3	ENSG00000128596	CCDC136	ENSG00000171435	KSR2
ENSG00000125510	OPRL1	ENSG00000206181	ELOA2	ENSG00000112981	NME5
ENSG00000180999	C1orf105	ENSG00000182950	ODF3L1	ENSG00000116661	FBXO2
ENSG00000213366	GSTM2	ENSG00000198929	NOS1AP	ENSG00000162571	TTLL10
ENSG00000063180	CA11	ENSG00000088543	C3orf18	ENSG00000152076	CCDC74B
ENSG00000162104	ADCY9	ENSG00000135423	GLS2	ENSG00000120262	CCDC170

ENSG	NAME
ENSG00000124194	GDAP1L1
ENSG00000132554	RGS22
ENSG00000174080	CTSF
ENSG00000205978	NYNRIN
ENSG00000147576	ADHFE1
ENSG00000261678	SCRT1
ENSG00000154153	RETREG1
ENSG00000170917	NUDT6
ENSG00000178150	ZNF114
ENSG00000124171	PARD6B
ENSG00000128536	CDHR3

ENSG	NAME
ENSG00000221989	OR2A2
ENSG00000214944	ARHGEF28
ENSG00000146054	TRIM7
ENSG00000203942	C10orf62
ENSG00000133256	PDE6B
ENSG00000196372	ASB13
ENSG00000267385	CTB-50L17.14
ENSG00000134533	RERG
ENSG00000164128	NPY1R
ENSG00000170500	LONRF2
ENSG00000066468	FGFR2

ENSG	NAME
ENSG00000170893	TRH
ENSG00000124249	KCNK15
ENSG00000113739	STC2
ENSG00000206013	IFITM5
ENSG00000175356	SCUBE2
ENSG00000149927	DOC2A
ENSG00000091831	ESR1

Supplementary Table 3: ERBB2 mutations in the RNAseq samples

**patient**	**cluster-nr**	**Consequence.HGVSp**	**mutType**	**AA-Change**	**domain**
p1	3	ENSP00000269571.4:p.D110E	SNV	D110E	ex
p2	3	ENSP00000269571.4:p.R190W	SNV	R190W	ex
p3	3	ENSP00000269571.4:p.D277Y	SNV	D277Y	ex
p4	3	ENSP00000269571.4:p.S310F	SNV	S310F	ex
p5	3	ENSP00000269571.4:p.S310F	SNV	S310F	ex
p6	1	ENSP00000269571.4:p.R678Q	SNV	R678Q	ex
p7	1	ENSP00000269571.4:p.L755S	SNV	L755S	TK
p8	3	ENSP00000269571.4:p.L755S	SNV	L755S	TK
p9	3	ENSP00000269571.4:p.V777L	SNV	V777L	TK
p10	3	ENSP00000269571.4:p.V777L	SNV	V777L	TK
p11	3	ENSP00000269571.4:p.-780_-781insGSP	InDel	780_-781insGSP	TK
p12	3	ENSP00000269571.4:p.I788F	SNV	I788F	TK
p13	3	ENSP00000269571.4:p.E876K	SNV	E876K	TK
p14	3	ENSP00000269571.4:p.M955I	SNV	M955I	TK
p15	3	ENSP00000269571.4:p.P983S	SNV	P983S	TK
p16	3	ENSP00000269571.4:p.E717D, ENSP00000269571.4:p.L755S	SNV	E717D, L755S	Ex/TK
p17	3	ENSP00000269571.4:p.L755S, ENSP00000269571.4:p.T862A	SNV	L755S, T862A	TK
p18	3	ENSP00000269571.4:p.D769Y, ENSP00000269571.4:p.E1114K	SNV	D769Y, E1114K	TK

ex: extracellular, TK: Tyrosine Kinase

Supplementary Table 4: IPA-Upstream Regulators using genes Suppl. Table 1

Upstream Regulator	Molecule Type	Predicted Activation State	Activation z-score	p-value of overlap	Target Molecules in Dataset
ERBB2	kinase	Activated	2.339	2.65E-05	ABCG2,ABL1,ABRACL,ACTB,AKT1,ATF4,BCL2,BCL3,CDC23,CDC42BPG,CEBPB,CX3CR1,DOK1,DUSP6,E2F3,EPHX2,ESR1,FFAR2,GALNT3,HES1,HES2,HGSNAT,HM13,IGF1R,IGF2R,IL6ST,ITGB6,KRT7,KRT81,LAD1,LMO4,LPAR2,LSS,MUCL1,MYCN,MYO10,NAGPA,NOTCH3,NPY1R,NRBP1,NUCB2,OPRL1,P2RY12,P2RY13,PDCD4,PHLDA2,PLAUR,POLK,POLR3GL,PRSS3,PTTG1IP,RETREG1,S100P,SDC1,SLC22A5,SMTN,SOX4,SPDEF,SYCP3,TGFBR1,TMEM132A,TNC,TSC22D1
RAF1	kinase	Activated	2.931	9.57E-05	BCL2,CD37,CDC42EP1,CTSL,DUSP4,DUSP6,ESR1,HNRNPAB,IGF1R,LAD1,MGLL,NQO1,PHLDA2,PLAUR,PPP1R10,PRSS3,SDC1,SLC26A2,TSC22D1,TUBB2A
ERG	transcription regulator	Activated	2.138	0.000156	ABCC4,ADGRG1,ARMC12,BCR,CDC42BPB,COLGALT1,DBN1,DIP2A,IGF1R,MAGI1,MAP3K5,MYCN,MYO10,PLAUR,PXN,RGS3,RRAS,SOX4,SREBF2,WASF3
NRG1	growth factor	Activated	2.154	0.000701	ARPC4,ATF1,ATF4,BCL2,BDNF,CDC42EP1,DUSP4,DUSP6,HES1,HK2,INSL3,LMO4,MARCKS,MGLL,MNX1,PDCD4,PGR,PLAUR,PXN,SOX4,TGFBR1
EGF	growth factor	Activated	2.563	0.000944	ABCG2,AKT1,B4GALT5,BCL2,CCL17,CDC42EP1,CEBPB,DGKD,DNMT3B,DUSP4,DUSP6,E2F3,ESR1,EZR,FAM89B,HES1,IGF1R,INSL3,MAP3K5,MNX1,MYCN,PDCD4,PGR,PHLDA2,PIK3R1,PLAUR,S100A9,SDC1,SLC37A1,SOX4,SPRY4,ST3GAL3,SYCP3,TFCP2L1,TNC,TRH,TUBB3,ULK1,VASP
TP73	transcription regulator	Activated	2.231	0.000953	AFAP1,ARVCF,BCAR1,BCL2,BID,CAMLG,DMTF1,EDA,FGFR2,G6PD,GLS2,GRAMD4,GTSE1,HAGH,HES1,HIVEP1,IGF1R,IGSF3,KCNK1,KLF5,LIMK1,MGMT,MYCN,NOTCH3,SHISA5,SPTBN4,TFCP2L1,TP53AIP1,TP53I11,UBE2D1,ULK1,WNK3
